# Cavity‐Compatible Light Extraction in Top‐Emitting Microcavity OLEDs via a Gentle Internal Corrugation Layer and Resonance‐Matched Narrowband Emission

**DOI:** 10.1002/advs.78013

**Published:** 2026-09-28

**Authors:** Young Rok Kim, Ajay Nimbalkar, Min Chul Suh

**Affiliations:** ^1^ Department of Information Display Kyung Hee University Seoul Dongdaemun‐Gu Republic of Korea

**Keywords:** internal outcoupling structures, light‐extraction strategies, microcavity OLEDs, narrowband emitters, top‐emitting OLEDs, viewing‐angle stability

## Abstract

Top‐emitting microcavity organic light‐emitting diodes (TE‐OLEDs) use a Fabry–Perot cavity to concentrate emission in the forward direction and narrow the electroluminescence spectrum, improving frontal efficiency and color purity. However, internal light extraction remains difficult because steep outcoupling textures can release confined optical modes while increasing local optical‐thickness variation, degrading step coverage, and perturbing the cavity‐defined angular and spectral response. Here, we introduce a gentle internal corrugation layer (GICL) as a cavity‐compatible light‐extraction structure for TE‐OLEDs and combine it with a tCzphB‐Fl‐based phosphor‐assisted thermally activated delayed fluorescence (TADF)‐sensitized fluorescence (pTSF) emissive system. GICL enhances the extraction of confined optical modes while reducing the risk of severe cavity perturbation associated with steep internal textures. The narrowband pTSF emissive system further reinforces the device‐level gain because a larger spectral fraction remains aligned with the microcavity resonance band. Under the same optical clear resin (OCR) condition, GICL increases the current efficiency from 302.7 to 378.4 cd/A, the power efficiency from 199.3 to 396.7 lm/W, and the external quantum efficiency from 34.8% to 69.2%, while reducing the angular color shift at 60° from Δu′v′ = 0.0197 to 0.0086. These results support GICL as a cavity‐compatible light‐extraction route for microcavity TE‐OLEDs.

## Graphical abstract

A gentle internal corrugation layer conformally reshapes the microcavity OLED stack, redistributing confined optical power toward extractable channels while preserving cavity‐controlled emission. Combined with optical clear resin and a resonance‐matched narrowband pTSF emissive system, this cavity‐compatible architecture delivers an external quantum efficiency of 69.2% with reduced angular color variation over wide viewing angles.

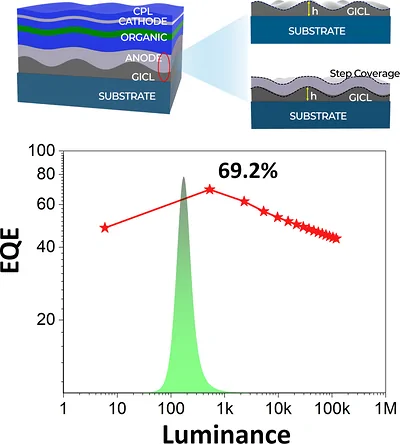

## 1 Introduction

Among active‐matrix organic light‐emitting diode (AMOLED) architectures, top‐emitting organic light‐emitting diodes (TE‐OLEDs) are widely used in high‐resolution displays because the emission is directed away from the substrate and the aperture ratio can be decoupled from the backplane layout [1, 2, 3, 4, 5, 6, 7, 8, 9, 10, 11, 12]. TE‐OLEDs also readily incorporate a Fabry–Perot microcavity, which narrows the electroluminescence (EL) spectrum and concentrates emission near the forward direction. These characteristics improve frontal efficiency and color purity, but they also increase viewing‐angle‐dependent color shift and make light‐extraction design more restrictive [13, 14, 15, 16, 17, 18].

A second‐order microcavity was adopted with consideration of fabrication reliability and the optical environment of the emitter. The thicker organic stack was selected to reduce susceptibility to particle‐related leakage and deposition nonuniformity, while the emitter–electrode separation was designed with consideration of the Purcell effect and coupling to metal‐related optical modes. The additional thickness was primarily allocated to the HTL, taking advantage of the favorable hole‐transport characteristics of the selected materials while limiting the ETL thickness and maintaining the intended cavity resonance. OLED multilayer structures can support confined optical channels, including waveguide (WG)‐ and surface plasmon polariton (SPP)‐related modes, whose relative contributions depend on the electrode configuration, multilayer optical thickness, emission wavelength, and cavity order. Improving only the outer optical interface is therefore insufficient because these internal loss channels cannot be fully recovered by controlling the air‐side interface alone. A practical light‐extraction strategy must address confined optical modes inside the device while maintaining the angular and spectral characteristics imposed by the microcavity [19, 20, 21, 22, 23, 24, 25, 26, 27, 28, 29, 30].

Conventional internal outcoupling structures based on wrinkles, corrugations, and strongly curved textures can enhance light extraction by scattering trapped optical modes. However, strong internal textures can also broaden the angular emission profile, disturb the narrow cavity‐shaped spectrum, and reduce deposition uniformity in the overlying layers [31, 32, 33, 34, 35, 36, 37]. The key geometric issue is local optical‐thickness variation across the cavity stack. When the underlying texture is steep, subsequently deposited layers are more likely to deviate from conformal coverage, producing spatial variation in the local resonance condition. Under this condition, stronger scattering does not necessarily lead to a useful device‐level gain because the cavity‐defined forward‐emission benefit can be weakened at the same time.

This trade‐off requires an internal structure that provides optical perturbation for trapped‐mode extraction while limiting local resonance distortion. In this work, we define a gentle internal corrugation layer (GICL) as an internal light‐extraction structure with a lower representative surface angle and broader lateral modulation than the steeper reference textures. The purpose of GICL is not to maximize scattering strength, but to provide cavity‐compatible light extraction by increasing the extraction of confined optical modes while mitigating the severe optical‐thickness variation associated with steep internal textures.

The device‐level benefit of such a structure also depends on the spectral properties of the emitter. In a microcavity device, only the spectral fraction overlapping with the microcavity resonance band contributes efficiently to cavity‐enhanced external output. Broad emitters retain a larger off‐resonant fraction and therefore undergo stronger spectral reshaping during cavity formation. By contrast, narrowband emitters retain a larger usable spectral fraction within the microcavity resonance band. In the present study, the narrowband emitter platform was implemented using a phosphor‐assisted thermally activated delayed fluorescence (TADF)‐sensitized fluorescence (pTSF) emissive system. Its emissive layer (EML) consists of DMIC‐TRZ as the TADF sensitizing host, Ir(ppy)_2_acac as the phosphorescent assistant, and tCzphB‐Fl as the terminal multi‐resonance TADF (MR‐TADF) emitter. In the previously reported pTSF strategy, the TADF host and phosphorescent assistant provide complementary sensitization pathways to the terminal emitter, combining efficient exciton utilization with narrowband emission. We employed this pTSF system to utilize the narrow emission of tCzphB‐Fl when investigating the effects of emitter bandwidth, microcavity resonance matching, and GICL‐induced optical extraction [38, 39, 40, 41].

Here, we introduce GICL as a cavity‐compatible light‐extraction structure for top‐emitting microcavity OLEDs and combine it with resonance‐matched narrowband emission. We examine the structural morphology and step coverage of GICL, compare its optical response with steeper reference structures using finite‐difference time‐domain analysis, and evaluate device performance under matched optical clear resin (OCR) conditions [42, 43, 44, 45, 46]. We further use polarization‐resolved angle‐dependent electroluminescence and emitter‐system comparison to assess whether the observed gain is consistent with enhanced extraction of confined optical modes and improved spectral overlap with the microcavity resonance band. These results support a design strategy in which internal geometry and emitter–microcavity spectral matching are jointly optimized for cavity‐compatible light extraction in TE‐OLEDs.

## 2 Results

### 2.1 Role of Emitter Bandwidth and Spectral Overlap in Microcavity Resonance Matching

In a Fabry–Perot microcavity, cavity‐assisted optical gain is effective mainly for spectral components that overlap with the microcavity resonance band. The device‐relevant gain is therefore limited by the microcavity resonance band, as discussed in Note S1. Emitter full width at half maximum (FWHM) is an important spectral parameter in this context because it affects how much of the original emission overlaps with the microcavity resonance band after cavity formation. A broad emitter contains a larger off‐resonant fraction outside the microcavity resonance band, whereas a narrowband emitter retains a larger fraction of its emission within the microcavity resonance band. This relationship was evaluated using the Bepp_2_: Ir(ppy)_2_acac phosphorescent system and the DMIC‐TRZ: Ir(ppy)_2_acac: tCzphB‐Fl pTSF system as representative broad and narrowband emission systems, respectively.

Here, **R** denotes a reference rather than a completely cavity‐free device. The ITO‐based **R** architecture was not intentionally tuned to a specific cavity order but can retain residual optical interference arising from the electrodes, refractive‐index contrasts, and multilayer optical thickness. **R1** and **R2** were used to establish the baseline responses of the phosphorescent and pTSF emitter systems, respectively, before introducing the intentionally designed Ag‐based microcavity and light‐extraction structures.

Figure 1a,b compares the electroluminescence spectra of the phosphorescent and pTSF devices under reference and microcavity conditions. In the phosphorescent system, the peak wavelength shifts from 521 to 534 nm and the FWHM narrows from 65 to 25 nm from **R1** to **P‐O1**. This large spectral change indicates that the cavity selects only a limited spectral fraction from the original broad emission band. In contrast, the pTSF system shows a smaller spectral change during cavity formation. From **R2** to **P‐O2**, the peak wavelength changes from 530 to 532 nm and the FWHM narrows only from 22 to 19 nm. This smaller spectral change suggests that a larger fraction of the narrowband emission already overlaps with the microcavity resonance band before cavity formation.

**FIGURE 1:**
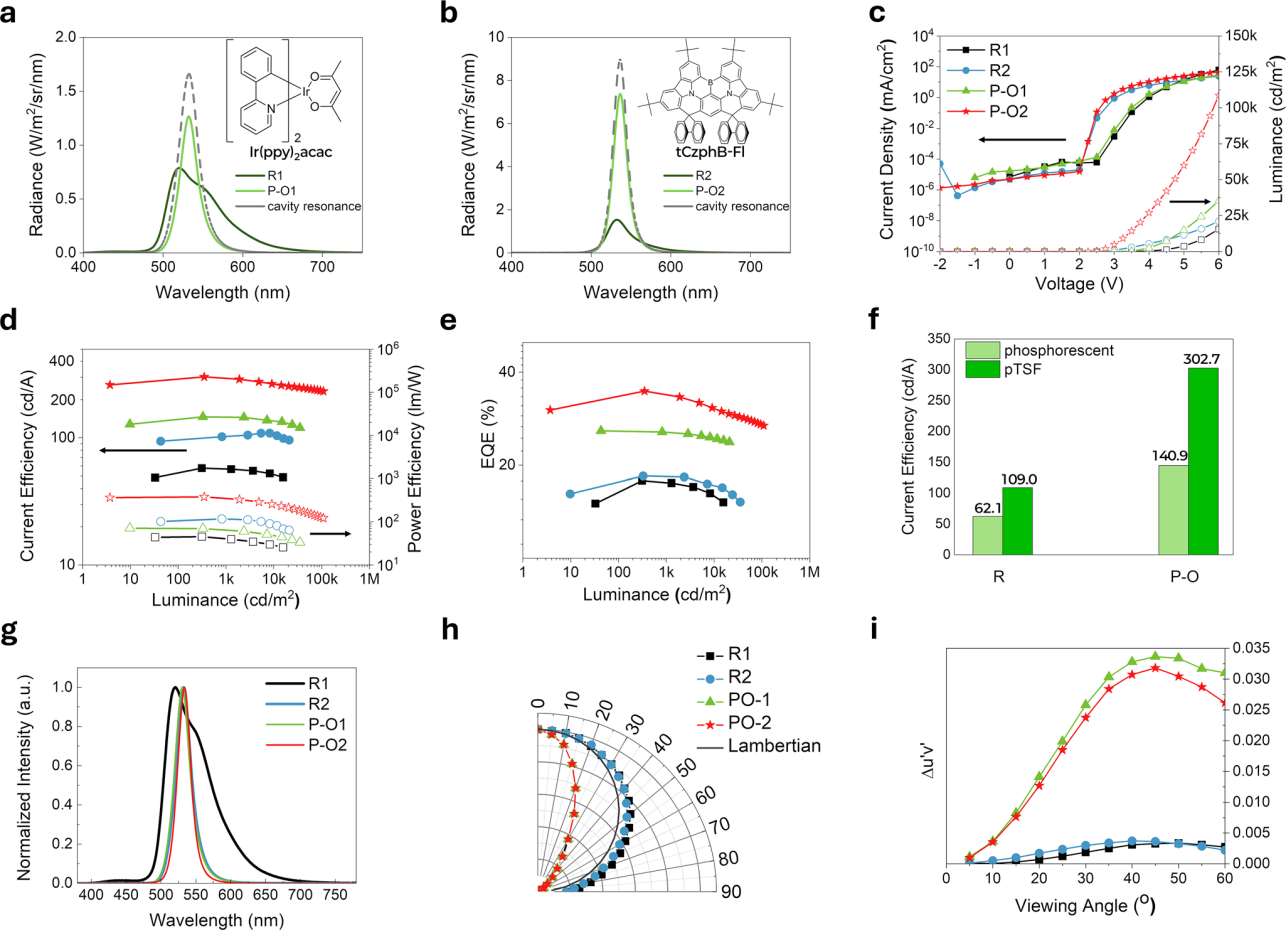
Role of emitter bandwidth and spectral overlap in microcavity resonance matching. (a,b) Radiance spectra of **R1** and **P‐O1** in the phosphorescent system and **R2** and **P‐O2** in the pTSF system, respectively, together with the corresponding cavity resonance. (c) Current density–voltage–luminance characteristics of **R1**, **R2**, **P‐O1**, and **P‐O2**. (d) Current efficiency and power efficiency as functions of luminance. (e) External quantum efficiency as a function of luminance. (f) Comparison of the maximum current efficiencies of the **R** and **P‐O** configurations in the phosphorescent and pTSF systems. (g) Normalized EL spectra measured at 0° for **R1**, **R2**, **P‐O1**, and **P‐O2**. (h) Angular luminance distributions of **R1**, **R2**, **P‐O1**, and **P‐O2** compared with the Lambertian distribution. (i) Angular color shifts (Δu′v′) of **R1**, **R2**, **P‐O1**, and **P‐O2**.

Figure 1c–f and Table 1 show how these differences in emitter system and spectral condition are reflected in device performance. Although Ir(ppy)_2_acac and tCzphB‐Fl exhibit relatively similar green emission peak wavelengths, their corresponding devices show different turn‐on voltages of 3.0 and 2.25 V, respectively. The emission peak represents the radiative transition energy of the final emitting species, whereas the turn‐on voltage is governed by the combined device‐level processes of charge injection, charge transport, carrier recombination, energy transfer, and conversion of the injected current into detectable luminance. The phosphorescent and pTSF devices employ different EML compositions, Bepp_2_: Ir(ppy)_2_acac and DMIC‐TRZ: Ir(ppy)_2_acac: tCzphB‐Fl, respectively, and the latter additionally involves a sensitized emission pathway terminating at tCzphB‐Fl. A related pTSF architecture has recently demonstrated narrowband emission with high external quantum efficiency and power efficiency, supporting pTSF as an efficient narrowband emissive platform. Therefore, the lower turn‐on voltage and higher current efficiency of the pTSF device cannot be inferred from its emission peak alone and are attributed to the combined electrical, photophysical, spectral, and angular‐emission characteristics of the different EML systems. The individual contributions of these factors were not independently isolated in the present study.

**TABLE 1:** Electrical and optical characteristics of reference and microcavity devices in phosphorescent and pTSF systems.

Device	Turn‐on voltage (V)	J (mA/cm^2^)^a^	Maximum efficiency			Peak λ (nm)^e^	FWHM(nm)^f^	Maximum luminance (cd/m^2^)	LCF^g^	CIE 1931 (x,y)^h^
			C.E (cd/A)^b^	P.E (lm/W)^c^	E.Q.E(%)^d^					
**R1**	3.0	62.8	62.1	63.9	19.4	521	65	15,603	1.1448 (± 2%)	(0.3264, 0.6188)
**R2**	2.25	25.1	109.0	117.0	26.1	530	22	20,946	1.1895 (± 2%)	(0.3068, 0.6664)
**P‐O1**	3.0	22.4	140.9	67.4	18.5	534	25	27,985	0.5306 (± 2%)	(0.2064, 0.7472)
**P‐O2**	2.25	46.6	302.7	199.3	34.8	532	19	108,659	0.5238 (± 2%)	(0.2008, 0.7473)

^a^ Current density at 6 V.

^b^ Current efficiency.

^c^ Power efficiency after Lambertian correction factor (LCF) correction.

^d^ External quantum efficiency after LCF correction.

^e^ Peak wavelength.

^f^ Full width at half maximum of the electroluminescence spectral intensity.

^g^ Lambertian correction factor obtained from JALS.

^h^ CIE 1931 color coordinates.

In the phosphorescent system, cavity formation increases the current efficiency from 62.1 to 140.9 cd/A and the power efficiency from 63.9 to 67.4 lm/W, whereas the external quantum efficiency changes from 19.4% to 5.5%. Thus, strong spectral reshaping does not necessarily produce a corresponding external quantum efficiency increase. In the pTSF system, the current efficiency increases from 109.0 to 302.7 cd/A, the power efficiency from 117.0 to 199.3 lm/W, and the external quantum efficiency from 26.1% to 34.8%. The maximum luminance also increases from 20,946 to 108,659 cd/m^2^ in the pTSF system, whereas it increases from 15,603 to 27,985 cd/m^2^ in the phosphorescent system.

These results indicate that the response to the **R‐to‐P‐O** device conversion depends not only on the introduction of the microcavity configuration but also on the spectral relationship between the emitter and the microcavity resonance band. In the phosphorescent system employing a Bepp_2_: Ir(ppy)_2_acac EML, the maximum current efficiency increases from 62.1 cd/A for **R1** to 140.9 cd/A for **P‐O1**, corresponding to a 2.27‐fold enhancement. In the narrowband pTSF system employing a DMIC‐TRZ: Ir(ppy)_2_acac: tCzphB‐Fl EML, the maximum current efficiency increases from 109.0 cd/A for **R2** to 302.7 cd/A for **P‐O2**, corresponding to a larger 2.78‐fold enhancement. Within each pair, the EML composition is maintained, and the principal intended change is the conversion from the R configuration to the corresponding P‐O microcavity configuration. Thus, the central comparison is the relative **R‐to‐P‐O** enhancement within each emitter system rather than the absolute efficiency difference between the two emitter systems.

The larger relative enhancement observed in the pTSF system is consistent with its narrower emission bandwidth and more favorable spectral overlap with the corresponding microcavity resonance band. From a spectral perspective, a smaller emitter FWHM may increase the fraction of emission that remains matched to the cavity resonance, allowing the cavity‐assisted optical gain to be translated more effectively into forward device output. However, the absolute efficiency difference between the phosphorescent and pTSF systems cannot be attributed to FWHM because the two systems also differ in their material composition and electrical and photophysical characteristics. The observed **R‐to‐P‐O** enhancement trend therefore suggests that emitter FWHM can be considered an important spectral factor in the response of an emitter system to microcavity formation, rather than establishing FWHM as the sole determinant of device efficiency. The detailed layer structures and thicknesses of **R1**, **R2**, **P‐O1**, and **P‐O2** are provided in Table S1. The normalized EL spectra, angular luminance distributions, and viewing‐angle‐dependent color‐coordinate variations of **R1**, **P‐O1**, **R2**, and **P‐O2** are shown in Figure 1g–i.

### 2.2 Surface Geometry Required for Cavity‐Compatible Internal Outcoupling

A light‐extraction structure for microcavity TE‐OLEDs must satisfy two requirements simultaneously. It must perturb confined WG‐ and SPP‐related optical modes inside the device, and it must do so without introducing excessive local optical‐thickness variation into the cavity stack. Figure 2a,b illustrates this geometric and optical requirement. When the surface profile changes gradually, the subsequently deposited layers can follow the underlying corrugation more conformally, which helps maintain a more uniform optical thickness across the cavity stack. When the profile is steep or abrupt, by contrast, conformal coverage becomes more difficult and the local resonance condition can vary more strongly across the device.

**FIGURE 2:**
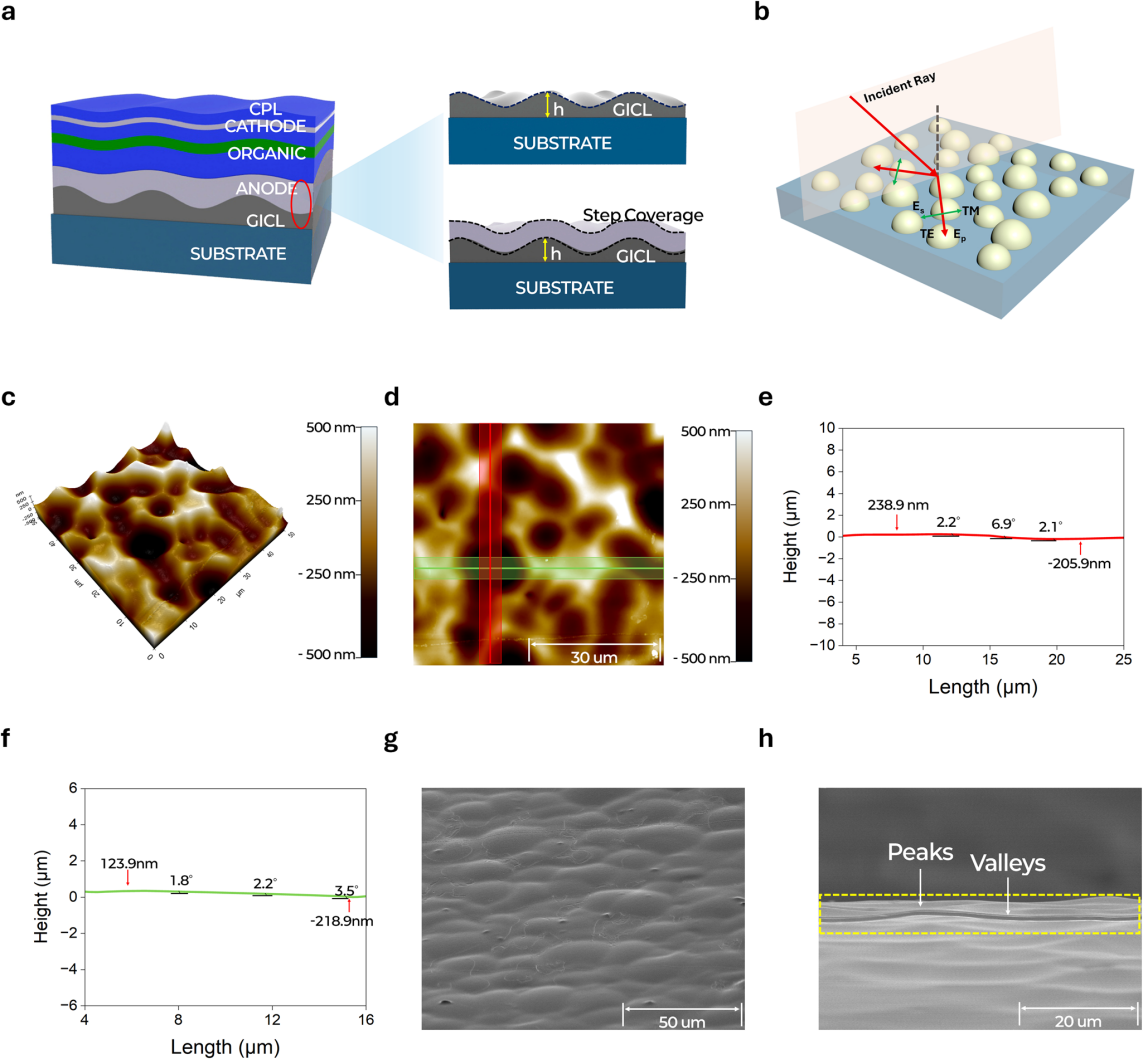
Surface morphology and conformal deposition of GICL. (a) Conformal deposition on GICL. (b) Schematic illustration of internal optical outcoupling by GICL. (c) Three‐dimensional atomic force microscopy (AFM) image. (d) Top‐view AFM image. (e) X‐direction line profile. (f) Y‐direction line profile. (g) Surface scanning electron microscopy (SEM) image. (h) Cross‐sectional SEM image.

The GICL surface exhibits microscale height modulation with a peak‐to‐valley height (R_pv_) of 1118.0 nm, a root‐mean‐square roughness (R_q_) of 144.5 nm, and an arithmetic‐average roughness (R_a_) of 116.6 nm over a 50 × 50 µm^2^ atomic force microscopy (AFM) scan area. Its representative surface angles are 2.1–6.9° and 1.8–3.5° along the x‐ and y‐direction profiles, respectively, and the corresponding representative peak‐to‐valley lateral spans are 8.88 and 8.87 µm. These parameters show that the measurable height modulation is distributed gradually over a micrometer‐scale lateral distance. The key geometric parameters of GICL and the reference outcoupling structures are comparatively summarized in Table S2a.

The reference structures clarify why roughness alone is insufficient for evaluating geometric severity in a microcavity stack. Wrinkle 1 has low roughness, with R_q_ = 20.6 nm, and low representative surface angles of 1.5° and 2.2°, but it functions as a shallow wrinkle reference rather than a microscale internal corrugation layer. Wrinkle 2 also has a modest R_q_ of 48.4 nm, but its representative surface angles increase to 55.7° and 53.1°, showing that low roughness amplitude does not necessarily correspond to gradual local geometry. The imprinted random microlens array (IRMLA) exhibits much larger topographic modulation, with R_q_ = 1359.0 nm, R_pv_ = 7867.0 nm, and a representative surface angle of 25.3°. These comparisons show that both roughness amplitude and representative surface angle should be considered when assessing whether an internal texture can be integrated into a microcavity stack without severe local geometric discontinuity.

The AFM and scanning electron microscopy (SEM) data in Figure 2c–h identify GICL as a continuous corrugated surface rather than a planar interlayer. The enlarged x‐ and y‐direction line profiles are provided in Figure S1. The corresponding profiles exhibit smooth, broadly sinusoidal‐like undulations; however, their nonuniform amplitudes and lateral spacings indicate that GICL should not be interpreted as an ideal periodic sinusoidal grating. Together with the roughness and angle analysis, these morphology data establish the structural basis of GICL as a gentle internal corrugation layer that provides measurable height modulation while avoiding abrupt local discontinuities.

The cross‐sectional SEM image in Figure 2h shows the deposited multilayer stack following the underlying GICL profile without severe local discontinuity. This conformal coverage is important because local discontinuities in the deposited stack can introduce spatial variation in optical thickness and local resonance condition. The cross‐sectional SEM comparison of **P2** and **G2** in Figure S2 further supports this structural interpretation. Therefore, Figures 2h and S2 are interpreted as structural evidence for multilayer conformality, not as direct optical evidence of cavity preservation. The geometric rationale linking internal texture geometry to possible local optical‐thickness variation is provided in Note S2.

The electrical and optical characteristics of the anode‐coated substrates are summarized in Table S2b. The average sheet resistances of the planar and GICL substrates are 0.3757 and 0.2453 Ω/sq, respectively, corresponding to calculated voltage drops of only 1.69 and 1.10 mV at 50 mA/cm^2^ for the 3 × 3 mm^2^ active pixel. Their average visible reflectances over 520–550 nm are 98.8% and 98.6%, respectively. These small differences in substrate series resistance and bottom‐anode reflectance cannot account for the observed device‐performance enhancement, supporting its association with GICL‐induced optical redistribution.

### 2.3 Structure‐Dependent Optical Extraction and Angular Emission Analysis

The finite‐difference time‐domain (FDTD) analysis in Figure 3 was used to examine whether internal corrugation in a microcavity device should be evaluated only by the increase in total extracted power or also by the angular form of the extracted output. A structure can increase total extracted power while still providing limited useful gain if the angular distribution broadens too strongly. The FDTD theory and simulation procedure are described in Note S3.

**FIGURE 3:**
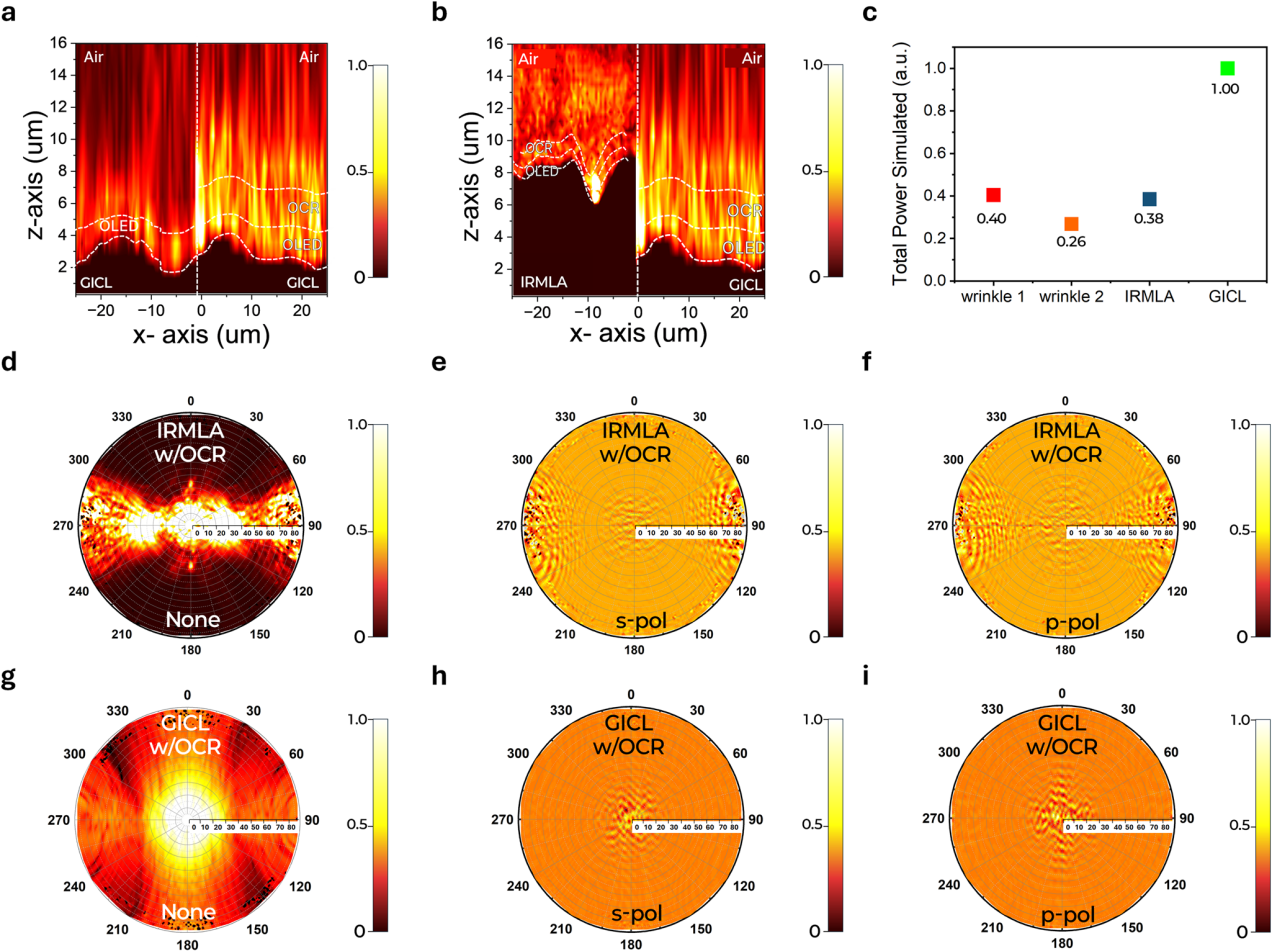
FDTD analysis of optical power extraction and far‐field emission in IRMLA‐ and GICL‐based microcavity OLEDs with OCR. (a,b) xz‐plane scattered electric‐field distributions of GICL‐ and IRMLA‐based structures. (c) Relative total simulated power for wrinkle, IRMLA, and GICL structures. (d–f) Far‐field emission distributions of the IRMLA‐based device for non‐polarized, s‐polarized, and p‐polarized components. (g–i) Far‐field emission distributions of the GICL‐based device for non‐polarized, s‐polarized, and p‐polarized components.

In this comparison, wrinkle 1 and wrinkle 2 serve as shallow and deep wrinkle references, respectively, IRMLA represents a steep large‐scale modulation, and GICL represents a gentle internal corrugation with gradual height variation and limited abrupt local discontinuities. The simulation was designed to compare these structure classes in terms of field redistribution, total extracted power, and forward‐directed optical output. The corresponding reference morphologies are provided in Figure S3, where Figure S3a,d,g shows the SEM images of wrinkle 1, wrinkle 2, and IRMLA, respectively, and Figure S3b,e,h shows their representative surface profiles and surface‐angle measurements.

OCR was introduced as an intermediate‐index auxiliary layer at the outer interface. The refractive index of OCR is 1.59, which lies between that of air and that of the organic‐side outcoupling structure. Because the extraction structure redistributes light over a broader angular range, the large refractive‐index discontinuity at the air interface would otherwise cause reflection‐related loss of the angularly scattered light. By inserting OCR between air and the extraction structure, this discontinuity is reduced and a larger fraction of the redistributed light can escape from the device. OCR therefore does not determine the internal redistribution itself but reduces the optical loss that occurs when the redistributed light reaches the outer interface. Figure 3a is consistent with this interpretation, showing stronger field extension toward the air region after OCR is introduced. The planar reference and the corresponding OCR‐induced far‐field change are provided in Figure S4 and S5.

Figure 3b compares the xz‐plane scattered electric‐field distributions of the IRMLA and GICL structures. The FDTD simulations were used as a geometry‐based optical analysis to compare how each surface structure redistributes and extracts optical power, rather than to model a specific EML or emitter system. IRMLA produces stronger local field perturbation, whereas GICL redistributes the field more gradually while still coupling optical power toward the outer region. This difference indicates that the optical consequence of internal corrugation depends on geometric severity rather than on the mere presence of scattering.

Figure 3c shows that GICL gives the highest relative total simulated power among the structures compared, followed by wrinkle 1, IRMLA, and wrinkle 2. Together with the structural references in Figure S3 and the roughness parameters in Table S2, this result shows that increasing depth and local slope does not produce a monotonic increase in extracted optical power. The deeper wrinkle reference gives lower transmitted power than the shallower wrinkle reference, and the steep IRMLA structure also remains below GICL. In this comparison, stronger perturbation alone is therefore not sufficient to improve the simulated optical response.

Complementary FDTD calculations were performed at 532 nm using identical dipole‐source conditions within a 30 nm EML. LEE was defined as the fraction of total dipole‐emitted optical power reaching the external air hemisphere. Under the same CPL and OCR conditions, the calculated LEE increases from 37.6% for **P‐O2** to 74.1% for **G‐O2**, corresponding to a 1.97‐fold enhancement. This comparison supports an additional light‐extraction contribution from GICL within the optical model.

The geometry‐dependent optical‐power trend in Figure 3c is consistent with the geometric rationale in Note S2. Structural corrugation can perturb confined optical components through local scattering and momentum redistribution, but the resulting extraction depends on the height and slope of the surface rather than on scattering strength alone. As summarized in Table S2, IRMLA exhibits a substantially larger peak‐to‐valley height of approximately 7.87 µm and a representative surface angle of approximately 25.3°, whereas GICL has a peak‐to‐valley height of 1.12 µm and gradual surface angles below 7°. The steep and strongly varying local surface normals of IRMLA therefore produce broader and less selective angular redirection.

The 3D ray‐tracing results in Figure S6 further clarify the consequence of this difference. Under identical simulation conditions, GICL directs 77.3% of the input power into the defined forward collection region, compared with 43.5% for IRMLA. The wide‐angle component increases from 11.4% for GICL to 43.6% for IRMLA. IRMLA also produces substantially more total‐internal‐reflection and back‐reflection events. Thus, stronger scattering in IRMLA does not directly represent absorptive loss, but instead reduces useful forward extraction by redirecting light toward extreme off‐axis, internally reflected, and backward‐propagating trajectories. GICL provides a more gradual optical perturbation that redistributes confined power while limiting excessive angular broadening and internal reflection.

Figure 3d–i further indicate why this distinction matters for a microcavity device. In the IRMLA structure, the non‐polarized, s‐polarized, and p‐polarized responses are broadly redistributed over angle and become more irregular, indicating strong scattering accompanied by substantial loss of directional control. In the GICL structure, angular redistribution also occurs, but the far‐field profiles are less broadly redistributed than in the IRMLA case. The corresponding far‐field comparison is provided in Figure S6.

OCR and GICL therefore play complementary optical roles. OCR reduces reflection‐related loss at the outer interface after light has been redistributed toward the air side, whereas the internal corrugation influences both the amount of scattered optical power and the angular form of that extracted output. In the present comparison, GICL shows the highest relative simulated power and less severe angular redistribution than the steep reference structures. These FDTD results provide the optical basis for the device‐level comparison in the following section, where the effects of GICL and OCR are evaluated using electrical and optical device characteristics.

### 2.4 Device performance of GICL microcavity OLEDs

GICL was introduced to improve optical extraction from microcavity TE‐OLEDs without relying on a steep internal texture. OCR was additionally applied to reduce outer‐interface optical loss associated with refractive‐index mismatch and unintended air‐gap formation during encapsulation. Within this device set, the transition from **P2** to **G2** reflects the effect of GICL without OCR, the transition from **G2** to **G‐O2** reflects the additional effect of OCR on the GICL structure, and the comparison between **P‐O2** and **G‐O2** provides a more direct assessment of the GICL effect under the same OCR condition. The nominal layer structures and thicknesses of **P2**, **P‐O2**, **G2**, and **G‐O2** are provided in Table S3.

The corresponding angle‐dependent normalized EL spectra are provided in Figure S7. Figure S8a,b examine whether GICL causes macroscopic emission non‐uniformity or pixel‐scale instability. The optical microscope images of the pixel region and the EL images of **P‐O2** and **G‐O2** were recorded at 1.75 mA/cm^2^. Both devices maintain stable pixel emission without severe local non‐uniformity. This observation indicates that the GICL structure does not break the emitting region into isolated bright spots or introduce obvious structural discontinuities at the pixel scale.

Figure 4a shows that **P2**, **P‐O2**, **G2**, and **G‐O2** exhibit the same turn‐on voltage of 2.25 V, and their current‐density characteristics remain broadly comparable over the measured driving range. The low‐bias region also does not show a clear increase in leakage current after introducing GICL. This trend is further supported by the current density at 6 V listed in Table 2, which changes only from 42.5 to 42.9 mA/cm^2^ from **P2** to **G2** and from 46.6 to 50.7 mA/cm^2^ from **P‐O2** to **G‐O2**. In Figure 4c, these current‐density changes are much smaller than the corresponding efficiency changes, especially the external quantum efficiency increase from 26.1% to 49.4% for **P2** to **G2** and from 34.8% to 69.2% for **P‐O2** to **G‐O2**. Therefore, the efficiency enhancement is not mainly explained by current‐flow variation or leakage current. It is more appropriately interpreted as an optical change associated with GICL‐induced extraction and OCR‐assisted outer‐interface outcoupling.

**FIGURE 4:**
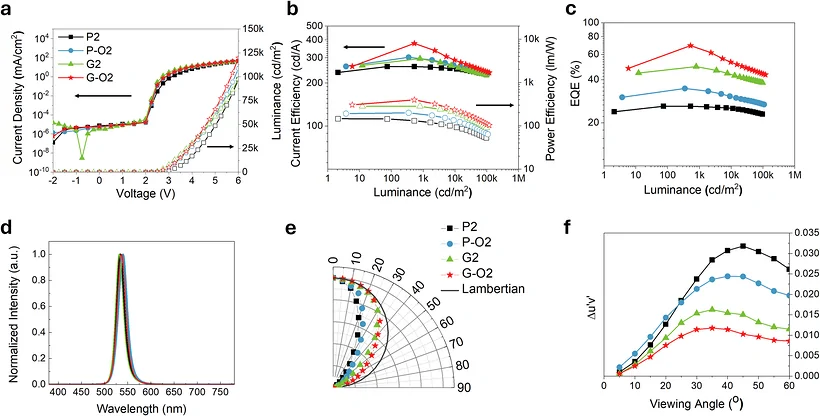
Device performance and angular emission characteristics of GICL microcavity OLEDs. (a) Current density‐voltage‐luminance characteristics of **P2**, **P‐O2**, **G2**, and **G‐O2**. (b,c) Current efficiency, power efficiency, external quantum efficiency, and luminance of **P2**, **P‐O2**, **G2**, and **G‐O2**. (d) Normalized EL spectra measured at 0° for **P2**, **P‐O2**, **G2**, and **G‐O2**. (e,f) Angular luminance distribution and angular color shift of **P2**, **P‐O2**, **G2**, and **G‐O2**.

**TABLE 2:** Electrical and optical characteristics of planar, OCR, GICL, and GICL/OCR devices in the pTSF microcavity system.

Device	Turn‐on voltage (V)	J (mA/cm^2^)^a^	Maximum efficiency			Peak λ (nm)^e^	FWHM(nm)^f^	Maximum luminance (cd/m^2^)	LCF^g^	CIE 1931 (x,y)^h^
			C.E (cd/A)^b^	P.E (lm/W)^c^	E.Q.E (%)^d^					
**P2**	2.25	42.5	260.7	145.8	26.1	534	19	97,174	0.4407 (± 2%)	(0.1987, 0.7634)
**P‐O2**	2.25	46.6	302.7	199.3	34.8	532	19	108,659	0.5238 (± 2%)	(0.2008, 0.7473)
**G2**	2.25	42.9	296.1	281.9	49.4	540	21	97,666	0.7576 (± 2%)	(0.2305, 0.7417)
**G‐O2**	2.25	50.7	378.4	396.7	69.2	537	22	119,889	0.8343 (± 2%)	(0.2271, 0.7459)

^a^ Current density at 6 V.

^b^ Current efficiency.

^c^ Power efficiency after LCF correction.

^d^ External quantum efficiency after LCF correction.

^e^ Peak wavelength.

^f^ Full width at half maximum of the electroluminescence spectral intensity.

^g^ Lambertian correction factor obtained from JALS.

^h^ CIE 1931 color coordinates.

The efficiency comparison supports this optical interpretation. As shown in Figure 4b, **G2** increases the external quantum efficiency from 26.1% to 49.4% relative to **P2**, indicating that the internal corrugation contributes to device output even before the addition of OCR. When OCR is added to the planar device, **P‐O2** reaches a current efficiency of 302.7 cd/A, a power efficiency of 199.3 lm/W, and an external quantum efficiency of 34.8%. When OCR is added to the GICL device, **G‐O2** reaches 378.4 cd/A, 396.7 lm/W, and 69.2%, respectively. Under the same OCR condition, the comparison between **P‐O2** and **G‐O2** shows that GICL increases the current efficiency by 75.7 cd/A, the power efficiency by 197.4 lm/W, and the external quantum efficiency by 34.4 percentage points. This matched comparison supports that GICL provides an additional extraction contribution under the same outer‐interface condition. Across five samples, **P‐O2** and **G‐O2** exhibited average maximum external quantum efficiency (EQE_max_) values of 34.2 ± 0.6% and 68.2 ± 0.7%, respectively, with limited variation among the measured samples. The EQE initially increases with current density, which is most likely associated with progressive trap filling and improved electron–hole balance. After reaching its maximum, the EQE decreases as the carrier and exciton densities increase, enhancing density‐dependent losses such as exciton–exciton annihilation and exciton–polaron quenching. At 10^3^ cd/m^2^, the relative EQE roll‐offs of **P‐O1**, **G‐O1**, **P‐O2**, and **G‐O2** are approximately 0.5%, 1.9%, 2.7%, and 4.6%, respectively. Thus, the GICL devices show moderately larger roll‐off than their corresponding planar devices. Because each P‐O/G‐O pair has the same EML composition and nominal organic multilayer structure, the larger roll‐off of **G‐O2** may be related to local current nonuniformity introduced by the corrugated internal geometry. A locally concentrated current and recombination distribution would increase the exciton and polaron densities and thereby enhance density‐dependent quenching.

Figure 4d shows that the 0°EL spectra of **P2**, **P‐O2**, **G2**, and **G‐O2** exhibit only minor spectral changes after introducing GICL and OCR. The efficiency enhancement is therefore not accompanied by severe spectral distortion, supporting the interpretation that the main change originates from optical extraction rather than spectral reshaping.

Figure 4e shows that **G‐O2** approaches a more Lambertian luminance distribution than **P‐O2**, and this angular redistribution is reflected in the Lambertian correction factor, which increases from 0.5238 to 0.8343. In Figure 4f, the angular color shift at 60° also decreases from Δu′v′ = 0.0197 to 0.0086. These results indicate that the device‐level gain is accompanied by a more balanced angular luminance distribution and reduced viewing‐angle‐dependent color variation.

The angle‐dependent normalized EL spectra of **P2**, **P‐O2**, **G2**, and **G‐O2** are provided in Figure S7, and the viewing‐angle EL images of the **G‐O2** pixel are provided in Figure S8c. These supporting data show that the GICL/OCR device maintains stable visible emission over the measured viewing angles. The device results are also consistent with the structural and FDTD analyses. GICL provides an internal corrugation pathway for optical extraction, while OCR reduces outer‐interface optical loss after light is redistributed toward the air side. The polarization‐resolved angular emission analysis in the following section further examines whether the gain is consistent with extraction of WG‐ and SPP‐related optical components. To assess the GICL‐induced extraction response under directly comparable conditions, **P‐O2** and **G‐O2** were compared because they have the same 30 nm pTSF EML, organic multilayer structure, Ag‐based microcavity, and OCR and differ only in the presence of GICL. The EQE_max_ increases from 34.76% for **P‐O2** to 69.23% for **G‐O2**, corresponding to a 1.99‐fold increase in EQE‐based extracted output. Because the internal quantum efficiency (IQE) was not independently determined, this value represents a relative device‐level extraction enhancement rather than an absolute light‐extraction efficiency.

### 2.5 Polarization‐Resolved Support for GICL‐Induced Light Extraction

Following the device‐level gain observed in Section 2.4, polarization‐resolved angular EL analysis was performed for **P‐O2** and **G‐O2** under the same OCR condition. A linear polarizer was used to separate the p‐ and s‐polarized far‐field components because these components respond differently to the optical modes confined within a microcavity stack. The p‐polarized component represents the transverse‐magnetic TM‐like far‐field response and can contain signatures associated with SPP‐related coupling, whereas the s‐polarized component represents the transverse‐electric TE‐like response and is more closely related to WG propagation. This polarization‐resolved analysis is not by itself a direct modal decomposition of WG and SPP modes, but it provides a mode‐sensitive experimental comparison of the GICL‐induced change in extracted emission.

Figure 5a–c shows the p‐polarized angular EL response of **P‐O2** and **G‐O2**. Compared with **P‐O2**, **G‐O2** exhibits higher angular EL intensity over the measured viewing‐angle range while maintaining a similar cavity‐shaped spectral profile. Because SPP modes at planar metal/organic interfaces are TM polarized, this enhancement is consistent with increased extraction or redistribution of TM‐like confined optical components that can include an SPP‐related contribution. However, the p‐polarized far‐field emission also contains radiative TM‐like cavity and waveguided components and therefore cannot be interpreted as a direct quantitative measurement of the SPP power fraction.

**FIGURE 5:**
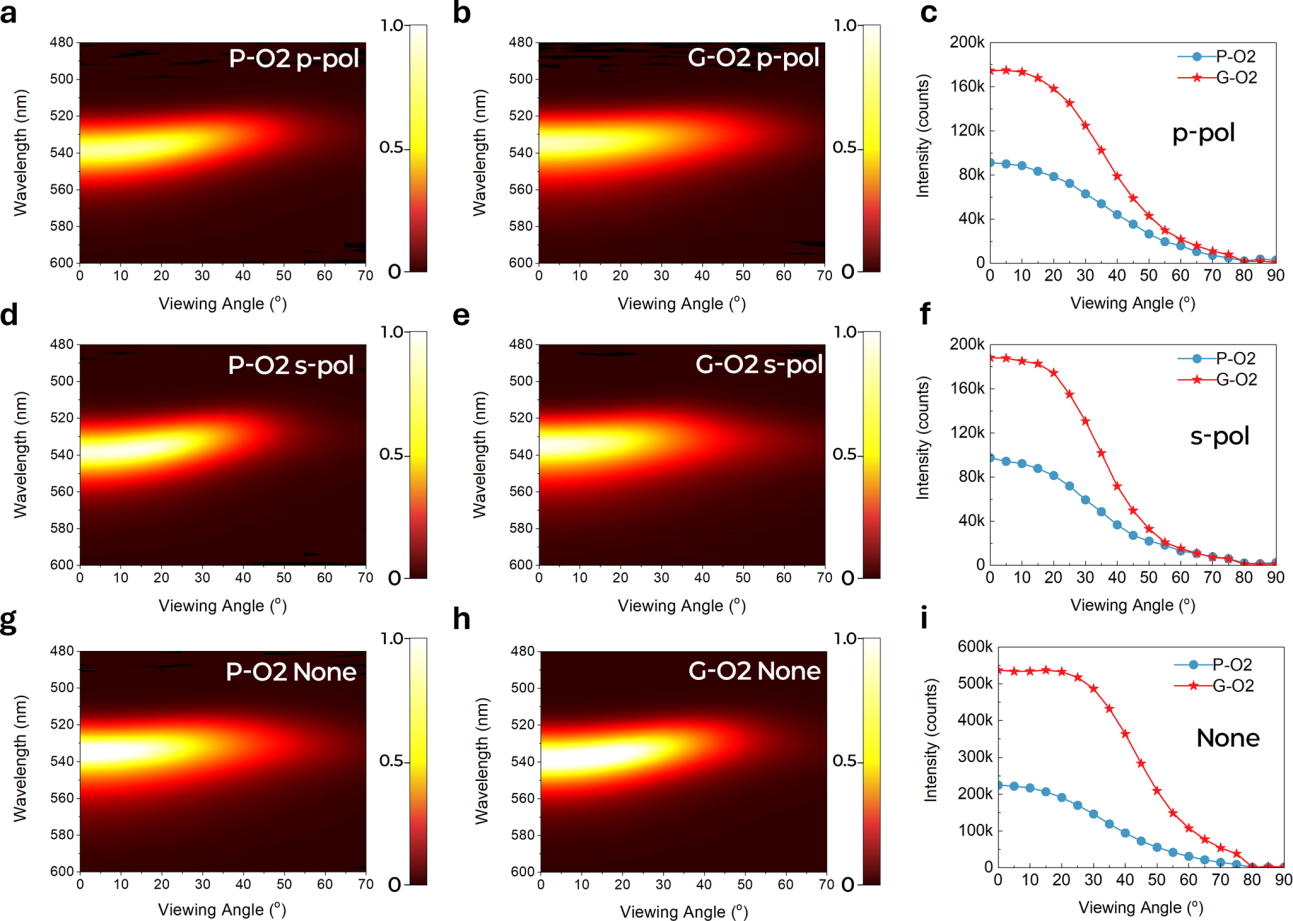
Polarization‐resolved angular emission analysis of **P‐O2** and **G‐O2** measured at 1.75 mA/cm^2^. (a,b) Angle‐resolved p‐polarized EL spectra of **P‐O2** and **G‐O2**, respectively. (c) Corresponding p‐polarized angular EL intensities. (d,e) Angle‐resolved s‐polarized EL spectra of **P‐O2** and **G‐O2**, respectively. (f) Corresponding s‐polarized angular EL intensities. (g,h) Angle‐resolved non‐polarized EL spectra of **P‐O2** and **G‐O2**, respectively. (i) Corresponding non‐polarized angular EL intensities.

Figure 5d–f shows the corresponding s‐polarized angular EL response. **G‐O2** again exhibits higher EL intensity than **P‐O2** over the measured angular range while maintaining a similar cavity‐controlled spectral profile. Since s‐polarized emission represents TE‐like optical components and does not couple directly to an SPP mode at a planar metal interface, its enhancement supports the extraction or redistribution of WG‐related and other TE‐like cavity‐confined components. The simultaneous enhancement under p‐ and s‐polarized detection indicates that the GICL‐induced gain is not confined to a single polarization channel.

Figure 5g–i provides the non‐polarized angular EL comparison. The non‐polarized EL intensity of **G‐O2** remains higher than that of **P‐O2** over the measured viewing‐angle range. This result confirms that the increases observed under p‐ and s‐polarized detection are accompanied by an increase in total extracted emission rather than arising solely from redistribution between the two polarization components.

Figure S9 provides the complementary wavelength‐ and in‐plane‐wavevector‐resolved optical‐mode distributions of **P‐O2** and **G‐O2**. Because the devices investigated here are top‐emitting OLEDs employing a reflective Al/Ag bottom anode, a substrate‐output channel equivalent to that of a bottom‐emitting OLED is not independently defined. The low‐u radiative components directed toward the external optical region are therefore treated together as the outcoupling‐related fraction, while the confined optical power is classified into the WG and SPP‐related high‐k regions, where u = k∥/k_0_. For **P‐O2**, the calculated outcoupling‐related, WG, and SPP‐related high‐k fractions are 29.7%, 40.0%, and 30.4%, respectively. For **G‐O2**, the corresponding fractions are 38.6%, 32.0%, and 29.4%, respectively. The outcoupling‐related fraction therefore increases by 30.0%, while the WG fraction decreases from 40.0% to 32.0% and the SPP‐related high‐k fraction decreases more modestly from 30.4% to 29.4%. The calculated redistribution is therefore dominated by recovery of WG‐related optical power, with a smaller change in the SPP‐related high‐k component. This result is consistent with the pronounced enhancement of the s‐polarized emission. The simultaneous increase in p‐polarized emission is also reasonable because the measured p‐polarized far‐field signal contains radiative TM‐like cavity and waveguided components in addition to any SPP‐related contribution.

The magnitude of the calculated optical redistribution is quantitatively consistent with the experimentally observed device‐output enhancement before Lambertian correction factor (LCF) correction. The current efficiency increases from 302.7 cd/A for **P‐O2** to 378.4 cd/A for **G‐O2**, corresponding to a 25.0% increase. Based on the reported EQE and LCF values, the J–V–L‐derived EQE before LCF correction is 34.8% divided by 0.5238, or 66.4%, for **P‐O2** and 69.2% divided by 0.8343, or 82.9%, for **G‐O2**. This corresponds to an increase of approximately 24.8%, which is comparable in magnitude to the 25.0% increase in current efficiency and the 30.0% increase in the simulated outcoupling‐related fraction.

The larger difference in the reported LCF‐corrected EQE, from 34.8% for **P‐O2** to 69.2% for **G‐O2**, results from applying the device‐specific LCF values that account for their different measured angular emission distributions. The LCF is a measurement correction and should not be interpreted as an additional GICL‐induced light‐extraction mechanism. The non‐polarized angular EL results in Figure 5g–i provides the experimental angular emission data associated with this correction. Taken together, the polarization‐resolved measurements, non‐polarized angular emission, and quantitative modal simulation consistently support that GICL enhances device output mainly by redistributing WG‐related optical power toward outcoupling‐related channels, with a smaller contribution from changes in the SPP‐related high‐k component.

### 2.6 Importance of Emitter FWHM in GICL‐Induced Device Performance

The device‐level response to GICL is not determined by internal geometry alone. As discussed in Section 2.1, microcavity optical gain depends on how much of the emitter spectrum overlaps with the microcavity resonance band after cavity formation. The same internal corrugation is therefore not expected to produce the same device response in broad and narrowband emitter systems. This point was examined by comparing planar and GICL devices in phosphorescent and pTSF microcavity systems. The detailed layer structures and thicknesses of **P‐O1**, **G‐O1**, **P‐O2**, and **G‐O2** are provided in Table S4.

Figure 6a–c and Table 3 show that GICL affects both emitter systems, but the magnitude of the device response differs substantially. In the phosphorescent system, the current efficiency increases from 140.9 cd/A in **P‐O1** to 163.0 cd/A in **G‐O1**. In the pTSF system, the corresponding increase is larger, from 302.7 cd/A in **P‐O2** to 378.4 cd/A in **G‐O2**. The same tendency is observed in the efficiency‐luminance and EQE‐luminance characteristics, where the separation between planar and GICL devices is smaller in the phosphorescent system and larger in the pTSF system. These results indicate that the same internal corrugation does not produce the same device‐level response in the two microcavity platforms.

**FIGURE 6:**
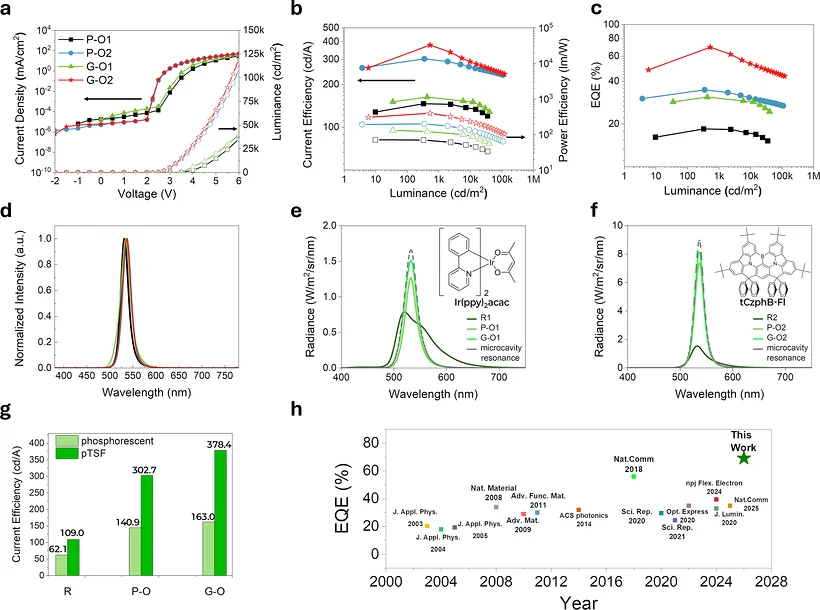
Importance of emitter FWHM in GICL‐induced device performance of microcavity OLEDs. (a–c) Current density‐voltage‐luminance characteristics, efficiency‐luminance characteristics, and EQE‐luminance characteristics of **P‐O1**, **G‐O1**, **P‐O2**, and **G‐O2**. (d) Normalized EL spectra measured at 0°. (e) Radiance spectra of **R1**, **P‐O1**, and **G‐O1** with the microcavity resonance band. (f) Radiance spectra of **R2**, **P‐O2**, and **G‐O2** with the microcavity resonance band. (g) Current efficiency comparison of **R**, **P‐O**, and **G‐O** structures. (h) External quantum efficiency summary of representative single‐stack green microcavity OLEDs.

**TABLE 3:** Electrical and optical characteristics of planar and GICL devices in phosphorescent and pTSF microcavity systems.

Device	Turn‐on voltage (V)	J (mA/cm^2^)^a^	Maximum efficiency			Peak λ (nm)^e^	FWHM(nm)^f^	Maximum luminance (cd/m^2^)	LCF^g^	CIE 1931 (x,y)^h^
			C.E (cd/A)^b^	P.E (lm/W)^c^	E.Q.E (%)^d^					
**P‐O1**	3.0	22.4	140.9	67.4	18.5	534	25	27,985	0.5306 (± 2%)	(0.2064, 0.7472)
**P‐O2**	2.25	46.6	302.7	199.3	34.8	532	19	108,659	0.5238 (± 2%)	(0.2008, 0.7473)
**G‐O1**	3.0	35.6	163.0	130.6	30.9	534	25	29,222	0.8264 (± 2%)	(0.2064, 0.7472)
**G‐O2**	2.25	50.7	378.4	396.7	69.2	537	22	119,889	0.8343 (± 2%)	(0.2271, 0.7459)

^a^ Current density at 6 V.

^b^ Current efficiency.

^c^ Power efficiency after LCF correction.

^d^ External quantum efficiency after LCF correction.

^e^ Peak wavelength.

^f^ Full width at half maximum of the electroluminescence spectral intensity.

^g^ Lambertian correction factor obtained from JALS.

^h^ CIE 1931 color coordinates.

Figure 6d–f provides spectral context for this difference. The normalized EL spectra measured at 0° show only minor spectral changes between the planar and GICL devices within each emitter system. Therefore, the different device response is not mainly associated with severe reshaping of the forward EL spectrum after introducing GICL. The more relevant difference appears in the radiance spectra. In the phosphorescent system, the broad emission band extends beyond the microcavity resonance band, so only a limited spectral fraction is efficiently reinforced by the cavity. In the pTSF system, a larger fraction of the narrowband emission remains aligned with the microcavity resonance band. Under this spectral condition, the larger GICL‐induced response observed in the narrowband system is consistent with more effective translation of the optical gain into the measured device characteristics.

Figure 6g,h further show that this emitter dependence remains visible in both current‐efficiency evolution and device‐level benchmarking. In the phosphorescent system, the current efficiency changes from 62.1 to 140.9 and then to 163.0 cd/A along the **R**, **P‐O**, and **G‐O** sequence. In the pTSF system, the corresponding sequence is 109.0, 302.7, and 378.4 cd/A. The increment from P‐O to G‐O is therefore 22.1 cd/A in the phosphorescent system and 75.7 cd/A in the pTSF system. Relative to the corresponding P‐O devices, these increments represent increases of approximately 15.7% and 25.0%, respectively. The larger relative **P‐O‐to‐G‐O** enhancement in the pTSF system is consistent with its narrower emission bandwidth and closer spectral alignment with the microcavity resonance band. However, because the two emitter systems also differ in EML composition and other electrical and photophysical characteristics, the difference in their GICL‐induced responses cannot be attributed exclusively to FWHM. The external quantum efficiency summary in Figure 6h further places the **G‐O2** device in the context of representative single‐stack green microcavity OLEDs reported in the literature.

The viewing‐angle‐dependent normalized EL spectra of **P2**, **P‐O2**, **G2**, and **G‐O2** are provided in Figure S7. The peak shifts measured from 0° to 60° decrease from approximately 13 nm for **P2** to 10 nm for **P‐O2**, 6 nm for **G2**, and 3 nm for **G‐O2**. These results confirm that the cavity emission remains angle dependent but that its observable angular spectral variation is progressively reduced after introducing GICL and OCR. The angular stability of **G‐O2** is therefore attributed to the combined effects of the narrow pTSF emission bandwidth, its overlap with the angle‐dependent cavity resonance, and the redistribution and extraction of confined optical power by GICL and OCR, rather than to emitter FWHM alone. The broader planar/GICL spectral comparison for both emitter systems is provided in Figure S10. The corresponding angular luminance distributions and color shifts are summarized in Figure S11, and viewing‐angle EL images of **G‐O2** are provided in Figure S8c. Figure S11c further compares the operational stability of **P‐O2** and **G‐O2** at an initial luminance of 1,000 cd/m^2^. The LT_95_ values (times to 95% of the initial luminance) are 111.3 h for **P‐O2** and 100.9 h for **G‐O2**, indicating comparable operational stability under the tested condition.

These results indicate that the device‐level consequence of GICL is closely related to emitter FWHM and spectral overlap with the microcavity resonance band. GICL does not determine the final device response independently of the emitter spectrum. Although the phosphorescent and pTSF systems differ in more than FWHM alone, the observed trend is consistent with the spectral‐overlap argument that a smaller FWHM leaves a larger fraction of emission within the microcavity resonance band. This condition allows the GICL‐induced optical gain to be converted more effectively into efficiency and luminance.

## 3 Conclusion

Microcavity TE‐OLEDs require internal light extraction without excessive disturbance of the cavity‐defined optical response. This requirement was addressed by introducing GICL as a gentle internal corrugation layer and combining it with OCR and a resonance‐matched narrowband emitter. GICL provides measurable internal height modulation with gradual height variation and limited abrupt local discontinuities, while OCR reduces outer‐interface optical loss after scattered light reaches the air side. This combination separates the role of internal optical redistribution from outer‐interface escape and provides a practical route for improving extraction in a microcavity device.

The GICL/OCR device based on the pTSF emissive system reached a current efficiency of 378.4 cd/A, a power efficiency of 396.7 lm/W, and an external quantum efficiency of 69.2%. Under the same OCR condition, the EQE increased from 34.8% in **P‐O2** to 69.2% in **G‐O2**, while the angular color shift at 60° decreased to Δu′v′ = 0.0086. The comparable current‐density characteristics and the limited change in the 0°EL spectra indicate that the efficiency enhancement is more appropriately interpreted as an optical extraction effect rather than a current‐flow or spectral‐reshaping effect. Polarization‐resolved angular EL analysis and the quantitative modal decomposition further indicate that the GICL‐induced gain is dominated by redistribution of WG‐related optical power toward outcoupling‐related channels, while the SPP‐related high‐k component changes more modestly.

The comparison between phosphorescent and pTSF microcavity systems shows that the device‐level response to GICL depends on emitter FWHM and spectral overlap with the microcavity resonance band. Although the two emitter systems differ in more than FWHM alone, the narrowband pTSF emissive system provides a more favorable spectral condition for translating GICL‐induced optical gain into device efficiency and luminance. These results indicate that internal corrugation geometry, outer‐interface index matching, and emitter‐resonance matching should be designed together for efficient microcavity TE‐OLEDs. High‐luminance efficiencies, EQE values before and after LCF correction, and five‐device reproducibility data are summarized in Table S5a–c.

## 4 Experimental Methods

### 4.1 OLED Fabrication

Planar glass, ITO‐coated glass, and externally supplied corrugated GICL substrates were sequentially cleaned by ultrasonication in acetone, isopropyl alcohol, and deionized water for 10 min. The corrugated GICL substrates were used as received without additional surface patterning or replication. All metal and organic layers were deposited by vacuum thermal evaporation under a base pressure of approximately 10^−^^7^ Torr.

For the reflective bottom electrode of the microcavity OLEDs, Al (50 nm) and Ag (50 nm) were sequentially deposited at 2 Å/s. The Al underlayer assisted the adhesion and continuous formation of Ag on the substrate and also formed part of the reflective electrode. The organic layers were deposited at rates of 0.5–2 Å/s. The semitransparent Ag cathode was deposited at 0.5 Å/s.

1,4,5,8,9,11‐Hexaazatriphenylenehexacarbonitrile (HAT‐CN) and *N,N*′‐bis(naphthalen‐1‐yl)‐*N,N*′‐bis(phenyl)benzidine (NPB) were used as the hole‐injection and hole‐transport materials, respectively. In the microcavity devices, repeated HAT‐CN/NPB layers were employed to provide the optical thickness required for cavity formation while maintaining carrier injection through the thick hole‐transport region. 4,4′,4″‐Tris(carbazol‐9‐yl)triphenylamine (TCTA) was inserted adjacent to the EML as a hole‐transporting and exciton‐confining layer.

The phosphorescent EML consisted of bis[2‐(2‐hydroxyphenyl)pyridinato]beryllium (Bepp_2_) as the host and bis(2‐phenylpyridine)(acetylacetonate)iridium(III) [Ir(ppy)_2_acac] as the phosphorescent emitter. The Ir(ppy)_2_acac concentration was 5 wt%, and the EML thickness was 30 nm. The pTSF EML consisted of 1,3‐dihydro‐1,1‐dimethyl‐3‐[3‐(4,6‐diphenyl‐1,3,5‐triazin‐2‐yl)phenyl]indeno[2,1‐b]carbazole (DMIC‐TRZ) as the TADF sensitizing host, Ir(ppy)_2_acac as the phosphorescent assistant, and tCzphB‐Fl as the terminal multi‐resonance TADF (MR‐TADF) emitter. The concentrations of Ir(ppy)_2_acac and tCzphB‐Fl were 10 and 2 wt%, respectively, and the total EML thickness was 30 nm.

4,7‐Diphenyl‐1,10‐phenanthroline (Bphen) was used as the electron‐transport layer. Lithium fluoride (LiF, 0.6 nm) and a thin Al layer (1 nm) were sequentially deposited as the electron‐injection layers. For the microcavity devices, Ag (20 nm) was subsequently deposited at 0.5 Å/s as the semitransparent cathode, followed by NPB (60 nm) as the optical capping layer. For the OCR‐containing devices, OCR with a refractive index of 1.59 was applied to the outer device surface at a nominal thickness of 1,000 nm to eliminate the interfacial air gap.

The complete device structures were as follows:

**R1**: Glass/ITO (150 nm)/HAT‐CN (15 nm)/NPB (40 nm)/TCTA (15 nm)/Bepp_2_: 5 wt% Ir(ppy)_2_acac (30 nm)/Bphen (30 nm)/LiF (0.6 nm)/Al (1 nm)/Al (100 nm).
**R2**: Glass/ITO (150 nm)/HAT‐CN (15 nm)/NPB (40 nm)/TCTA (15 nm)/DMIC‐TRZ: 10 wt% Ir(ppy)_2_acac: 2 wt% tCzphB‐Fl (30 nm)/Bphen (30 nm)/LiF (0.6 nm)/Al (1 nm)/Al (100 nm).
**P‐O1**: Planar glass/Al (50 nm)/Ag (50 nm)/HAT‐CN (7 nm)/NPB (75 nm)/HAT‐CN (7 nm)/NPB (75 nm)/TCTA (15 nm)/Bepp_2_: 5 wt% Ir(ppy)_2_acac (30 nm)/Bphen (42 nm)/LiF (0.6 nm)/Al (1 nm)/Ag (20 nm)/NPB (60 nm)/OCR (1,000 nm).
**G‐O1**: Corrugated GICL substrate/Al (50 nm)/Ag (50 nm)/HAT‐CN (7 nm)/NPB (75 nm)/HAT‐CN (7 nm)/NPB (75 nm)/TCTA (15 nm)/Bepp_2_: 5 wt% Ir(ppy)_2_acac (30 nm)/Bphen (42 nm)/LiF (0.6 nm)/Al (1 nm)/Ag (20 nm)/NPB (60 nm)/OCR (1,000 nm).
**P2**: Planar glass/Al (50 nm)/Ag (50 nm)/HAT‐CN (7 nm)/NPB (75 nm)/HAT‐CN (7 nm)/NPB (75 nm)/TCTA (15 nm)/DMIC‐TRZ: 10 wt% Ir(ppy)_2_acac: 2 wt% tCzphB‐Fl (30 nm)/Bphen (42 nm)/LiF (0.6 nm)/Al (1 nm)/Ag (20 nm)/NPB (60 nm).
**P‐O2**: Planar glass/Al (50 nm)/Ag (50 nm)/HAT‐CN (7 nm)/NPB (75 nm)/HAT‐CN (7 nm)/NPB (75 nm)/TCTA (15 nm)/DMIC‐TRZ: 10 wt% Ir(ppy)_2_acac: 2 wt% tCzphB‐Fl (30 nm)/Bphen (42 nm)/LiF (0.6 nm)/Al (1 nm)/Ag (20 nm)/NPB (60 nm)/OCR (1,000 nm).
**G2**: Corrugated GICL substrate/Al (50 nm)/Ag (50 nm)/HAT‐CN (7 nm)/NPB (75 nm)/HAT‐CN (7 nm)/NPB (75 nm)/TCTA (15 nm)/DMIC‐TRZ: 10 wt% Ir(ppy)_2_acac: 2 wt% tCzphB‐Fl (30 nm)/Bphen (42 nm)/LiF (0.6 nm)/Al (1 nm)/Ag (20 nm)/NPB (60 nm).
**G‐O2**: Corrugated GICL substrate/Al (50 nm)/Ag (50 nm)/HAT‐CN (7 nm)/NPB (75 nm)/HAT‐CN (7 nm)/NPB (75 nm)/TCTA (15 nm)/DMIC‐TRZ: 10 wt% Ir(ppy)_2_acac: 2 wt% tCzphB‐Fl (30 nm)/Bphen (42 nm)/LiF (0.6 nm)/Al (1 nm)/Ag (20 nm)/NPB (60 nm)/OCR (1,000 nm).

### 4.2 Corrugated Substrate and Device Fabrication

The corrugated substrate used to form the GICL was provided by an external supplier and used as received. The OLED electrode and subsequent organic multilayer stack were deposited directly onto the supplied corrugated surface.

### 4.3 Optical Simulation Analysis

Optical mode analysis and optical power‐fraction calculations were performed using SETFOS to evaluate the optical behavior of the pristine microcavity top‐emitting OLED stacks. The simulations were used to examine the dependence of optical mode distribution on hole‐transport layer (HTL) and electron‐transport layer (ETL) thickness, the power‐dissipation spectrum as a function of normalized in‐plane wavevector and wavelength, and the outcoupled radiance of the reference microcavity devices. The emission wavelength was swept from 380 to 700 nm in 1 nm steps. The simulated layer structures followed the microcavity OLED stacks used in this work.

### 4.4 FDTD simulations

Three‐dimensional finite‐difference time‐domain simulations were performed using commercial software (Lumerical Solutions Inc.) to analyze optical power extraction and far‐field emission from planar, wrinkle, IRMLA, and GICL‐based microcavity OLED structures. The simulation geometries were constructed from measured surface‐profile data obtained from the morphological characterization of the outcoupling structures. Emitting dipoles oriented along the x, y, and z directions were placed in the emitting layer to represent isotropic emission. The z direction was defined normal to the substrate surface. The refractive indices of all materials used in the simulations were based on experimentally determined values obtained from ellipsometry. Near‐field distributions, relative total simulated power, and far‐field emission profiles were extracted from the simulations.

### 4.5 OLED Characterization

Current density‐voltage‐luminance characteristics were measured using *a J–V–L* measurement system equipped with a spectroradiometer (CS‐2000, Konica Minolta). External quantum efficiency, current efficiency, power efficiency, electroluminescence spectra, and CIE 1931 color coordinates were measured using the same spectroradiometric system with a Keithley 2400 source meter. Luminous intensity and Lambertian correction factors were measured using a JALS system (JooAm Co.). The Lambertian correction factor (LCF) for each device was obtained from the JALS measurement. The reported LCF‐corrected EQE and power efficiency were calculated by multiplying the corresponding values obtained from the *J–V–L* system by the experimentally determined device‐specific LCF. All planar and GICL devices were characterized using the same projected emitting area of 3 mm × 3 mm. Although the corrugated GICL geometry increases the actual microscopic interfacial area, the current density and luminance of both device configurations were calculated using the identical projected pixel area.

### 4.6 Surface Morphology and Roughness Analysis

Surface morphologies of the films and devices were examined using an ultra‐high‐resolution field‐emission scanning electron microscope (UHR FE‐SEM, Hitachi S‐4700) operated at an accelerating voltage of 10 kV at Kyung Hee University, Seoul, Republic of Korea. Atomic force microscopy measurements were carried out using an XE‐100 AFM system (Park Systems) to analyze surface topography, roughness, and line profiles.

### 4.7 Optical Microscopy

Optical microscope images of the devices were obtained using an Olympus BX51M metallurgical microscope equipped with a U‐LH100HG lamp and U‐RFL‐T power supply to evaluate pixel morphology and emission uniformity.

## Author Contributions

**Young Rok Kim**: methodology, conceptualization, investigation, validation, formal analysis, data curation, visualization, writing – original draft, writing – review and editing. **Ajay Nimbalkar**: methodology, software, formal analysis, validation, writing – original draft, writing – review and editing. **Min Chul Suh**: conceptualization, supervision, funding acquisition, project administration, resources, writing – review and editing.

## Funding

This work was supported by the National Research Foundation of Korea (NRF) grant funded by the Korean government (MSIT) (RS‐2026‐25542348) and by the Technology Innovation Program (RS‐2025‐25452683, RS‐2026‐25526873, and RS‐2026‐25529892) funded by the Ministry of Trade, Industry and Resources (MOTIR, Korea) through the Korea Planning & Evaluation Institute of Industrial Technology (KEIT). This research is partially funded by the BK21 FOUR program of Graduate School, Kyung Hee University (20240380).

## Conflicts of Interest

The authors declare no conflicts of interest.

## Supporting information




**Supporting File**: advs78013‐sup‐0001‐SuppMat.docx.

## Data Availability

The data that support the findings of this study are available from the corresponding author upon reasonable request.
