# Supplementary material for: Cavity‐Compatible Light Extraction in Top‐Emitting Microcavity OLEDs via a Gentle Internal Corrugation Layer and Resonance‐Matched Narrowband Emission
Source: Adv Sci (Weinh). 2026 Sep 28:e78013. Online ahead of print. doi: 10.1002/advs.78013 (PMC13618774; doi:10.1002/advs.78013)
Supplement: Supplementary file 1 [file ADVS-9999-e78013-s001.docx]

Supporting Information

Title

Cavity-Compatible Light Extraction in Top-Emitting Microcavity OLEDs via a Gentle Internal Corrugation Layer and Resonance-Matched Narrowband Emission

Young Rok Kim, Ajay Nimbalkar, and Min Chul Suh*

Department of Information Display, Kyung Hee University, Seoul 02447, Republic of Korea
E-mail: [mcsuh@khu.ac.kr](mailto:mcsuh@khu.ac.kr)

**Table of Contents**

**Table S1.** Device structures and layer thicknesses of reference and microcavity devices employing phosphorescent and pTSF emissive systems on planar glass substrates.

**Table S2.** Geometric, electrical, and optical characteristics of the GICL and reference substrates.

**Figure S1. a** x-direction line profile before enlargement. The black box indicates the enlarged region shown in **Figure 2e**. **b** y-direction line profile before enlargement. The black box indicates the enlarged region shown in **Figure 2f**.

**Figure S2.** Cross-sectional SEM images of the planar **P2** and GICL-based **G2** devices.

**Figure S3.** Surface morphology, representative surface profiles, and xz-plane scattered electric-field distributions of wrinkle and IRMLA structures with OCR. **a** Surface SEM image of wrinkle 1. **b** Representative surface profile of wrinkle 1. **c** xz-plane scattered electric-field distribution of wrinkle 1 with OCR. **d** Surface SEM image of wrinkle 2. **e** Representative surface profile of wrinkle 2. **f** xz-plane scattered electric-field distribution of wrinkle 2 with OCR. **g** Surface SEM image of IRMLA. **h** Representative surface profile of IRMLA. **i** xz-plane scattered electric-field distribution of IRMLA with OCR.

**Figure S4.** FDTD analysis of optical power extraction and far-field emission in microcavity OLEDs with OCR. xz-plane scattered electric-field distributions of microcavity OLED structures.

**Figure S5.** FDTD analysis of optical power extraction and far-field emission in microcavity OLEDs with OCR. **a–c** Far-field emission distributions of the microcavity OLED device for non-polarized, s-polarized, and p-polarized components. **d–f** Far-field emission distributions of the microcavity OLED with OCR device for non-polarized, s-polarized, and p-polarized components.

**Figure S6.** Simulated far-field emission and ray-propagation comparison of IRMLA- and GICL-based microcavity OLEDs with OCR. **a–c** Simulated far-field emission distributions of the IRMLA-based structure for non-polarized, s-polarized, and p-polarized components, respectively. **d–f** Corresponding far-field emission distributions of the GICL-based structure. **g, h** Receiver-plane irradiance maps obtained from 3D non-sequential ray tracing using the measured IRMLA and GICL surface geometries, respectively. **i, j** Representative ray-propagation trajectories for the IRMLA and GICL structures, respectively. The ray-tracing analysis was performed at a wavelength of 550 nm using 10,000 incident rays. The results show that the steep IRMLA geometry produces stronger wide-angle redirection, internal reflection, and backward propagation, whereas the gradual GICL geometry directs a larger fraction of optical power toward the forward collection region.

**Table S3.** Device structures and layer thicknesses of planar, OCR, GICL, and GICL/OCR devices in the pTSF microcavity system.

**Figure S7.** Angle-dependent normalized EL spectra of **P2**, **P-O2**, **G2**, and **G-O2** in the pTSF microcavity system. **a** **P2**. **b** **P-O2**. **c** **G2**. **d** **G-O2**.

**Figure S8. a, b** Optical microscope images and EL images of **P-O2** and **G-O2** recorded at 1.75 mA/cm². **c** EL images of the **G-O2** pixel at viewing angles of 0°, 20°, 40°, and 60°.

**Figure S9.** Wavelength-dependent optical-mode distributions of **P-O2** and **G-O2**. **a, b** Calculated optical-power distributions as functions of wavelength and normalized in-plane wavevector (u = k∥/k₀) for **P-O2** and **G-O2**, respectively. The outcoupling-related, WG, and SPP-related high-k regions are indicated.

**Figure S10.** Viewing-angle-dependent normalized EL spectra of planar and GICL devices in phosphorescent and pTSF microcavity systems. **a** **P-O1**. **b** **G-O1**. **c** **P-O2**. **d** **G-O2**.

**Figure S11.** Angular emission characteristics and operational stability of **P-O2** and **G-O2**. **a** Angular luminance distributions. **b** Angular color shift (Δu′v′) as a function of viewing angle. **c** Operational lifetime characteristics measured at an initial luminance of 1,000 cd/m². The LT₉₅ values are 111.3 h for **P-O2** and 100.9 h for **G-O2**.

**Table S4.** Device structures and layer thicknesses of planar and GICL devices in phosphorescent and pTSF microcavity systems.

**Table S5a.** Maximum efficiencies and efficiencies at luminances of 1,000 and 10,000 cd/m² for all devices.

**Table S5b.** External quantum efficiencies before and after application of the Lambertian correction factor (LCF).

**Table S5c.** Device-to-device reproducibility of the maximum external quantum efficiency for **P-O2** and **G-O2** (n = 5).

**Supporting Note S1.** Optical Cavity Effect and Angular Emission Characteristics

**Supporting Note S2.** Geometric Rationale for Cavity-Compatible Gentle Internal Corrugation

**Supporting Note S3.** FDTD Simulation Architecture and Scope

**Table S1**. Device structures and layer thicknesses of reference and microcavity devices employing phosphorescent and pTSF emissive systems on planar glass substrates.

|  | **Thickness (nm)** | |
| --- | --- | --- |
|  | **R1** | **R2** |
| **Al** | 100 | 100 |
| **LiF/Al** | 0.6/1 | 0.6/1 |
| **Bphen** | 30 | 30 |
| **EML** | Bepp_2_: Ir(ppy)_2_acac(5%) 30 | DMIC-TRZ: Ir(ppy)_2_acac (10%): tCzphB-Fl (2%) 30 |
| **TCTA** | 15 | 15 |
| **NPB** | 40 | 40 |
| **HATCN** | 15 | 15 |
| **ITO** | 150 | 150 |
| **Glass Substrate** | 2,000 | 2,000 |

|  | **Thickness (nm)** | |
| --- | --- | --- |
|  | **P-O1** | **P-O2** |
| **OCR** | 1,000 | 1,000 |
| **CPL (NPB)** | 60 | 60 |
| **Ag** | 20 | 20 |
| **LiF/Al** | 0.6/1 | 0.6/1 |
| **Bphen** | 42 | 42 |
| **EML** | Bepp_2_: Ir(ppy)_2_acac(5%) 30 | DMIC-TRZ: Ir(ppy)_2_acac(10%): tCzphB-Fl (2%) 30 |
| **TCTA** | 15 | 15 |
| **NPB** | 75 | 75 |
| **HATCN** | 7 | 7 |
| **NPB** | 75 | 75 |
| **HATCN** | 7 | 7 |
| **Al/Ag** | 50/50 | 50/50 |
| **Glass Substrate** | 2,000 | 2,000 |

**Table S2.** Geometric, electrical, and optical characteristics of the GICL and reference substrates.

**a** AFM-derived geometric parameters

|  | **GICL** | **wrinkle 1** | **wrinkle 2** | **IRMLA** |
| --- | --- | --- | --- | --- |
| **R_pv_ (nm)** | 1118.0 | 86.4 | 194.0 | 7867.0 |
| **R_q_ (nm)** | 144.5 | 20.6 | 48.4 | 1359.0 |
| **R_a_ (nm)** | 116.6 | 17.6 | 41.6 | 1076.0 |
| **Representative surface angle (°)** | x: 2.1–6.9; y: 1.8–3.5 | 1.5, 2.2 | 55.7, 53.1 | 25.3 |
| **Available lateral descriptor** | Peak-to-valley span: 8.88 µm (x), 8.87 µm (y) | Approximate pitch: 80 nm | Approximate pitch: 150–200 nm | Approximate pitch: 50 µm |

**R_pv_, R_q_**, and **R_a_** denote the peak-to-valley height, root-mean-square roughness, and arithmetic average roughness, respectively.

**b** Electrical and optical characteristics of the substrates after anode deposition

| **Substrate structure** | **Measured sheet resistance, Rₛ (mΩ/sq)** | **Average Rₛ (Ω/sq)** | **Active pixel area (mm²)** | **Calculated voltage drop at 50 mA/cm² (mV)** | **Average visible reflectance, R (%, 520–550 nm)** |
| --- | --- | --- | --- | --- | --- |
| **Bare glass** | N/A | N/A | N/A | N/A | N/A |
| **Planar substrate + anode** | 350, 387, 390 | 0.3757 | 3 × 3 (9.0) | 1.69 | 98.80 |
| **GICL substrate + anode** | 213, 288, 235 | 0.2453 | 3 × 3 (9.0) | 1.10 | 98.60 |
| **Wrinkle reference + anode** | 410, 435, 422 | 0.4223 | 3 × 3 (9.0) | 1.90 | 97.40 |
| **IRMLA reference + anode** | 520, 580, 550 | 0.5500 | 3 × 3 (9.0) | 2.48 | 97.20 |

**Figure S1.** **a** x-direction line profile before enlargement. The black box indicates the enlarged region shown in **Figure 2e**. **b** y-direction line profile before enlargement. The black box indicates the enlarged region shown in **Figure 2f**.

**Figure S2.** **a** Cross-sectional SEM image of the planar device (**P2**). **b** Cross-sectional SEM image of the GICL device (**G2**).

**Figure S3.** Surface morphology, representative surface profiles, and xz-plane scattered electric-field distributions of wrinkle and IRMLA structures with OCR. **a** Surface SEM image of wrinkle 1. **b** Representative surface profile of wrinkle 1. **c** xz-plane scattered electric-field distribution of wrinkle 1 with OCR. **d** Surface SEM image of wrinkle 2. **e** Representative surface profile of wrinkle 2. **f** xz-plane scattered electric-field distribution of wrinkle 2 with OCR. **g** Surface SEM image of IRMLA. **h** Representative surface profile of IRMLA. **i** xz-plane scattered electric-field distribution of IRMLA with OCR.

**Figure S4.** FDTD analysis of optical power extraction and far-field emission in microcavity OLEDs with OCR. xz-plane scattered electric-field distributions of microcavity OLED structures.

**Figure S5.** FDTD analysis of optical power extraction and far-field emission in microcavity OLEDs with OCR. **a–c** Far-field emission distributions of the microcavity OLED device for non-polarized, s-polarized, and p-polarized components. **d–f** Far-field emission distributions of the microcavity OLED with OCR device for non-polarized, s-polarized, and p-polarized components.

**Figure S6.** Simulated far-field emission and ray-propagation comparison of IRMLA- and GICL-based microcavity OLEDs with OCR. **a–c** Simulated far-field emission distributions of the IRMLA-based structure for non-polarized, s-polarized, and p-polarized components, respectively**. d–f** Corresponding far-field emission distributions of the GICL-based structure. **g, h** Receiver-plane irradiance maps obtained from 3D non-sequential ray tracing using the measured IRMLA and GICL surface geometries, respectively. **i, j** Representative ray-propagation trajectories for the IRMLA and GICL structures, respectively. The ray-tracing analysis was performed at a wavelength of 550 nm using 10,000 incident rays. The results show that the steep IRMLA geometry produces stronger wide-angle redirection, internal reflection, and backward propagation, whereas the gradual GICL geometry directs a larger fraction of optical power toward the forward collection region.

**Table S3**. Device structures and layer thicknesses of planar, OCR, GICL, and GICL/OCR devices in the pTSF microcavity system

|  | **Thickness (nm)** | | | |
| --- | --- | --- | --- | --- |
|  | **P2** | **P-O2** | **G2** | **G-O2** |
| **OCR** | - | 1,000 | - | 1,000 |
| **CPL (NPB)** | 60 | 60 | 60 | 60 |
| **Ag** | 20 | 20 | 20 | 20 |
| **LiF/Al** | 0.6/1 | 0.6/1 | 0.6/1 | 0.6/1 |
| **Bphen** | 42 | 42 | 42 | 42 |
| **EML DMIC-TRZ: Ir(ppy)_2_acac(10%):  tCzphB-Fl(2%)** | 30 | 30 | 30 | 30 |
| **TCTA** | 15 | 15 | 15 | 15 |
| **NPB** | 75 | 75 | 75 | 75 |
| **HATCN** | 7 | 7 | 7 | 7 |
| **NPB** | 75 | 75 | 75 | 75 |
| **HATCN** | 7 | 7 | 7 | 7 |
| **Al/Ag** | 50/50 | 50/50 | 50/50 | 50/50 |
| **Substrate** | 2,000  (Planar glass substrate) | 2,000  (Planar glass substrate) | 2,000  (GICL substrate) | 2,000  (GICL substrate) |

**Figure S7.** Angle-dependent normalized EL spectra of **P2**, **P-O2**, **G2**, and **G-O2** in the pTSF microcavity system measured at viewing angles from 0° to 60°. **a** **P2**. **b P-O2**. **c** **G2**. **d** **G-O2**. The corresponding peak shifts are approximately 13, 10, 6, and 3 nm, respectively.

**Figure S8. a, b** Optical microscope images and EL images of **P-O2** and **G-O2** recorded at 1.75 mA/cm²**. c** EL images of the **G-O2** pixel at viewing angles of 0°, 20°, 40°, and 60°.

**Figure S9.** Wavelength-dependent optical-mode distributions of **P-O2** and **G-O2**. **a, b** Calculated optical-power distributions as functions of wavelength and normalized in-plane wavevector (u = k∥/k₀) for **P-O2** and **G-O2**, respectively. The outcoupling-related, WG, and SPP-related high-k regions are indicated.

**Figure S10.** Viewing-angle-dependent normalized EL spectra of planar and GICL devices in phosphorescent and pTSF microcavity systems. **a P-O2. b G-O2. c P-O2. d G-O2**.

**Figure S11.** Angular emission characteristics and operational stability of **P-O2** and **G-O2**. **a** Angular luminance distributions. **b** Angular color shift (Δu′v′) as a function of viewing angle. **c** Operational lifetime characteristics measured at an initial luminance of 1,000 cd/m². The LT₉₅ values are 111.3 h for **P-O2** and 100.9 h for **G-O2**.

**Table S4**. Device structures and layer thicknesses of planar and GICL devices in phosphorescent and pTSF microcavity systems

|  | **Thickness (nm)** | | | |
| --- | --- | --- | --- | --- |
|  | **P-O1** | **G-O1** | **P-O2** | **G-O2** |
| **OCR** | 1,000 | 1,000 | 1,000 | 1,000 |
| **CPL (NPB)** | 60 | 60 | 60 | 60 |
| **Ag** | 20 | 20 | 20 | 20 |
| **LiF/Al** | 0.6/1 | 0.6/1 | 0.6/1 | 0.6/1 |
| **Bphen** | 42 | 42 | 42 | 42 |
| **EML** | Bepp_2_: Ir(ppy)_2_acac(5%) 30 | Bepp_2_: Ir(ppy)_2_acac(5%) 30 | DMIC-TRZ : Ir(ppy)_2_acac(10%) : tCzphB-Fl(2%) 30 | DMIC-TRZ : Ir(ppy)_2_acac(10%) : tCzphB-Fl(2%) 30 |
| **TCTA** | 15 | 15 | 15 | 15 |
| **NPB** | 75 | 75 | 75 | 75 |
| **HATCN** | 7 | 7 | 7 | 7 |
| **NPB** | 75 | 75 | 75 | 75 |
| **HATCN** | 7 | 7 | 7 | 7 |
| **Al/Ag** | 50/50 | 50/50 | 50/50 | 50/50 |
| **Substrate** | 2,000  (Planar glass substrate) | 2,000  (Planar glass substrate) | 2,000  (GICL substrate) | 2,000  (GICL substrate) |

**Table S5a.** Maximum efficiencies and efficiencies at luminances of 1,000 and 10,000 cd/m² for all devices.

| **Device** | **Current efficiencyᵃ** | | | **Power efficiencyᵇ** | | | **External quantum efficiencyᶜ** | | |
| --- | --- | --- | --- | --- | --- | --- | --- | --- | --- |
|  | **Max** | **1,000** | **10,000** | **Max** | **1,000** | **10,000** | **Max** | **1,000** | **10,000** |
| **R1** | 62.1 | 61.2 | 55.3 | 63.9 | 57.2 | 40.4 | 19.4 | 19.1 | 17.3 |
| **P-O1** | 140.9 | 140.2 | 130.6 | 67.4 | 61.2 | 45.6 | 18.5 | 18.4 | 17.2 |
| **G-O1** | 163.0 | 159.7 | 151.1 | 130.6 | 113.9 | 90.1 | 30.9 | 30.3 | 28.7 |
| **R2** | 109.0 | 102.6 | 106.2 | 117.0 | 116.2 | 85.5 | 26.1 | 25.8 | 24.7 |
| **P2** | 260.7 | 259.3 | 251.5 | 145.8 | 124.6 | 94.5 | 26.1 | 26.0 | 25.3 |
| **P-O2** | 302.7 | 294.6 | 265.0 | 199.3 | 183.2 | 132.2 | 34.8 | 33.8 | 30.5 |
| **G2** | 296.1 | 293.2 | 260.2 | 281.9 | 275.5 | 198.5 | 49.4 | 49.0 | 43.5 |
| **G-O2** | 378.4 | 360.6 | 288.9 | 396.7 | 364.2 | 231.9 | 69.2 | 66.0 | 53.0 |

**Table S5b.** External quantum efficiencies before and after application of the Lambertian correction factor (LCF).

| **Device** | **EQE before LCF correction (%)** | **LCF** | **LCF-corrected EQE (%)** |
| --- | --- | --- | --- |
| **R1** | 16.9 | 1.1448 | 19.4 |
| **R2** | 21.9 | 1.1895 | 26.1 |
| **P-O1** | 34.9 | 0.5306 | 18.5 |
| **P-O2** | 66.4 | 0.5238 | 34.8 |
| **P2** | 59.2 | 0.4407 | 26.1 |
| **G2** | 65.2 | 0.7576 | 49.4 |
| **G-O1** | 37.4 | 0.8264 | 30.9 |
| **G-O2** | 82.9 | 0.8343 | 69.2 |

**Table S5c.** Device-to-device reproducibility of the maximum external quantum efficiency for **P-O2** and **G-O2** (n = 5).

| **Device** | **Individual EQEₘₐₓ values (%)** | **n (number of devices)** | **Average EQEₘₐₓ ± standard deviation (%)** |
| --- | --- | --- | --- |
| **P-O2** | 34.8, 34.7, 34.4, 33.8, 33.3 | 5 | 34.2 ± 0.6 |
| **G-O2** | 69.2, 67.7, 68.0, 68.6, 67.7 | 5 | 68.2 ± 0.7 |

ᵃ Current efficiency (cd/A). ᵇ Power efficiency (lm/W). ᶜ External quantum efficiency after Lambertian correction (%).

**Supporting Note** **S1.** Optical Cavity Effect and Angular Emission Characteristics

Most modern smartphones, tablets, and laptops adopt a front-emission configuration based on top-emitting OLEDs with microcavity structures. This design is particularly effective for enhancing on-axis luminance and achieving high color purity due to the constructive interference effect inside the optical cavity. The Fabry–Pérot microcavity, formed between the reflective bottom electrode and the semi-transparent top electrode, supports multi-beam interference that selectively amplifies emission at specific wavelengths.

The resonance condition for constructive interference is described by Equation (S1.1):

$2ndcos\theta=m\lambda$ (S1.1)

where n is the refractive index, d is the effective cavity thickness, *θ* is the viewing angle, and m is the interference order. As *θ* increases, cos*θ* decreases, resulting in a shift of λ toward shorter wavelengths, typically perceived as blue shifting.

The full intensity distribution as a function of wavelength λ and emission angle θ is given by Equation (S1.2), which incorporates multiple reflective interactions and their phase relationships:

$I\left( \lambda, \theta\right)=\frac{T_{t}\left[ 1+R_{b}+2\sqrt{R_{b}}\cos(-\phi_{b}+\frac{4\pi z\cos\left( \theta_{EML} \right)}{\lambda}) \right]}{\left( 1-\sqrt{R_{b}R_{t}} \right)^{2}+4\sqrt{R_{b}R_{t}}{sin}^{2}(\frac{\Delta\emptyset}{2})}I_{0}(\lambda)$ (S1.2)

Here, Rb and Rt are the reflectivity of the bottom and top mirrors, Tt is the transmittance, φb is the reflection phase, and z denotes the optical path length from the reflective electrode to the emission zone. This expression predicts the angular dependence of spectral output by accounting for phase-sensitive interference.

The spectral linewidth of the emission is governed by the finesse of the cavity, represented by the full width at half maximum (FWHM), as expressed in Equation (S1.3):

$FWHM=\frac{\lambda^{2}}{2L_{cav}} \times\frac{1-\sqrt{R_{b}R_{t}}}{\pi\sqrt[4]{R_{b}R_{t}}}$ (S1.3)

This relation indicates that stronger reflectors (higher R) yield narrower spectra but exacerbate angular sensitivity.

Lastly, the emission wavelength λ(θ) under oblique viewing conditions can be derived from a multilayer phase condition, shown in Equation (S1.4):

$\lambda\left( \theta\right)=\frac{4\pi\left( \sum n_{i}d_{i} \right)\cos\left( \theta\right)}{\emptyset\left( \theta\right)-2\pi m}$ (S1.4)

This equation models the combined effect of multiple thin-film layers (nᵢ, dᵢ) and their associated phase shifts, emphasizing how angle-dependent interference fundamentally alters the OLED spectral output.

These theoretical foundations underscore both the strength and limitation of the microcavity approach: while enhancing spectral purity and efficiency along the normal direction, it introduces significant angle-dependent emission variance, motivating the need for additional angular control strategies.

The conversion from the CIE 1931 color space to the CIE 1976 color space is done to create a more perceptually uniform color space. This means that the distance between two colors in the CIE 1976 space is more representative of the perceived difference between those two colors to the human eye.

The conversion process involves transforming the CIE 1931 (x, y) chromaticity coordinates to the CIE 1976 (u', v') chromaticity coordinates. This transformation is performed using Equations (S1.5) and (S1.6):

$$u^{'}=\frac{4x}{-2x+12y+3} (S1.5)$$

$$v^{'}=\frac{9y}{-2x+12y+3} (S1.6)$$

The maximum peak wavelength shift observed between 0° and 70° is quantified by calculating the color difference (Δu′v′) in the CIE 1976 chromaticity space. This color difference is calculated as the Euclidean distance between the color coordinates at two different measurement angles using Equation (S1.7):

$\Delta u^{'}v^{'}= \sqrt{{(v{}_{0}^{'}-v_{x}')}^{2}+{(u{}_{0}^{'}-u_{x}')}^{2}}$ (S1.7)

In this equation, (u′,v′) represent the color coordinates. The subscripts 0 and x denote the measurement angles, where (u′,v′) are the coordinates at the 0° angle and (ux′,vx′) are the coordinates at the angle of measurement, x.

**Supporting Note S2.** Geometric Rationale for Cavity-Compatible Gentle Internal Corrugation.

This note provides a geometric rationale for evaluating the gentle internal corrugation layer (GICL) in a microcavity OLED. The purpose is not to calculate a spatially resolved microcavity resonance map, but to clarify why the local geometry of an internal texture can influence the cavity response after multilayer deposition.

For a simplified multilayer cavity, the one-way optical thickness can be expressed as

$$D = \Sigmaᵢ nᵢdᵢ (S2.1)$$

where **D** is the one-way optical thickness, and **nᵢ** and **dᵢ** are the refractive index and physical thickness of layer **i**, respectively. The microcavity resonance wavelength is related to the optical thickness of the cavity stack. Therefore, local changes in deposited layer thickness can change the local optical thickness and can shift the local resonance condition.

In a first-order approximation, this sensitivity can be expressed as

$$\frac{\Delta\lambdaᵣₑₛ}{\lambdaᵣₑₛ} \approx\frac{\Delta D}{D} (S2.2)$$

where **ΔD** is a local change in optical thickness and **Δλᵣₑₛ** is the corresponding change in resonance wavelength. This relation is used only to indicate the expected sensitivity of the microcavity resonance to optical-thickness variation. It is not used here to calculate an absolute local resonance shift.

The geometric severity of an internal texture can be described using the representative surface angle obtained from measured line profiles:

$$\thetaₛ = tan⁻¹(|\frac{\Delta z}{\Delta x} |) (S2.3)$$

where θₛ is the representative surface angle, **Δz** is the height change, and **Δx** is the corresponding lateral distance. This angle is used as a practical descriptor of local geometry. A larger representative surface angle can make conformal deposition more difficult, but the relation between surface angle and local optical-thickness variation is not assumed to be one-to-one.

The term “gentle” refers to a corrugated morphology with measurable height modulation, gradual height variation, and limited abrupt discontinuities, rather than the lowest roughness or a flat surface. The GICL surface has an RMS roughness of 144.5 nm over a 50 × 50 µm area, indicating that it is not a planar interlayer. However, the line profiles in **Figure 2e, f** show gradual height variation rather than abrupt local steps. The wrinkle and IRMLA reference structures in Figure S4 provide comparison cases with different roughness amplitudes and representative surface angles.

This note therefore supports the structural rationale that GICL can reduce the risk of severe local optical-thickness variation compared with steeper internal textures. However, it should not be read as a rigorous proof of local resonance uniformity or complete cavity preservation. Direct verification of local resonance uniformity would require additional spatially resolved optical-thickness or resonance mapping.

**Supporting Note S3.** FDTD Simulation Architecture and Scope

3D Finite-Difference Time-Domain (FDTD) calculations were performed using Ansys Lumerical (v2025 R2.2) to evaluate the optical near-field distribution and far-field outcoupling behavior of planar and corrugated microcavity OLEDs.

Geometry and material stack: The complete layout was organized within a Full OLED Stack structure group containing a representative GICL profile constructed from measured surface-profile data, the functional OLED organic and electrode layers, OCR, and the encapsulation glass. The representative profile preserved the measured peak-to-valley height and local slope characteristics used for the geometry-based comparison.

Dipole emitters and power distribution: Three mutually orthogonal electric dipole sources oriented along the x, y, and z directions were positioned in the EML. Their incoherently summed response was used to represent isotropic emission.

Power normalization: A closed three-dimensional transmission monitor surrounding the dipole group was used to integrate the emitted Poynting-vector flux. Metallic absorption was evaluated using the advanced absorption analysis group.

Spatial monitoring and sampling: Orthogonal two-dimensional frequency-domain monitors were used to record spatial field distributions, and a local mesh smaller than 5 nm was applied near the corrugated metal/organic interfaces. Dipole positions were sampled at representative crest, slope, and trough locations. The calculations were not repeated over multiple independent surface realizations; accordingly, they are used to compare geometry-dependent optical trends rather than to establish a statistical millimeter-scale average.

| **Object Name** | **Type in Lumerical** | **Physical Role in Simulation** |
| --- | --- | --- |
| **Full OLED Stack** | Structure Group | Parent group housing GICL, OLED, OCR, and Encapsulation layers. |
| **GICL** | Structure Group | Representative profile based on measured surface morphology. |
| **Dipole** | Layout Group | Houses 3 orthogonal dipoles (source_1, 1_1, 1_1_1) for isotropic averaging. |
| **trans_box** | Analysis Group | Closed 3D Poynting monitor box around dipoles to measure absolute Ptotal. |
| **pabs_adv** | Analysis Group | Absorption box calculating power dissipation (Pabs) in metal electrodes. |
| **XY, XZ_1, YZ** | DFTMonitors | 2D frequency-domain monitors capturing electric field heatmaps along orthogonal cuts. |
| mesh | Mesh Override | Refined spatial grid (<5 nm) at corrugated organic/metal boundaries. |

1. Theoretical Framework

The optical properties of the OLED devices were investigated using the three-dimensional finite-difference time-domain (3D FDTD) method, a full-wave electromagnetic simulation technique that directly solves Maxwell’s curl equations in the time domain. This approach enables accurate modeling of light–matter interactions in multilayer and nanostructured systems.

The governing Maxwell’s equations in differential form are:

$$\nabla\times\mathbf{E}\left( \mathbf{r},t \right)=-\mu\frac{\partial\mathbf{H}\left( \mathbf{r},t \right)}{\partial t} \text{(S}\text{3}\text{.}\text{1}\text{)}$$

$$\nabla\times\mathbf{H}\left( \mathbf{r},t \right)=\epsilon\frac{\partial\mathbf{E}\left( \mathbf{r},t \right)}{\partial t}+\mathbf{J}\left( \mathbf{r},t \right) \text{(S}\text{3}\text{.}\text{2}\text{)}$$

where **E** and **H** denote the electric and magnetic fields, respectively, while ε and μ represent the permittivity and permeability of the medium. The current density term **J** represents the excitation source.

In the FDTD method, space and time are discretized using the **Yee grid**, where electric and magnetic field components are staggered both spatially and temporally. The fields are updated iteratively using finite-difference approximations:

$$E^{n+1}=E^{n}+\frac{\Delta t}{\epsilon}(\nabla\times H)^{n} \text{(S}\text{3}\text{.}\text{3}\text{)}$$

$$H^{n+1}=H^{n}-\frac{\Delta t}{\mu}(\nabla\times E)^{n} \text{(S}\text{3}\text{.}\text{4}\text{)}$$

where $\Delta t$is the time step determined by the Courant stability condition.

2. Dipole Source Representation of OLED Emission

To model spontaneous emission in OLEDs, point dipole sources were embedded within the emissive layer. These dipoles simulate radiative recombination of excitons and were oriented along three orthogonal directions (x, y, z) to account for isotropic emission.

The power radiated by an oscillating dipole is given by:

$$P=\frac{\mu_{0}\omega^{4}\mid\mathbf{p}\mid^{2}}{12\pi c} \text{(S}\text{3}\text{.}\text{5}\text{)}$$

where:

- $\mathbf{p}$is the dipole moment
- $\omega$is the angular frequency
- $c$is the speed of light

Dipole orientation plays a crucial role in coupling to optical modes, particularly surface plasmon polaritons (SPPs) and waveguided modes.

3. Optical Mode Distribution in OLEDs

The total emitted optical power in OLEDs is distributed into several channels:

$$P_{total}=P_{air}+P_{substrate}+P_{waveguide}+P_{SPP} \text{(S}\text{3}\text{.}\text{6}\text{)}$$

where:

- $P_{air}$: Extracted light into air (useful emission)
- $P_{substrate}$: Light trapped in substrate modes
- $P_{waveguide}$: Light confined within organic layers
- $P_{SPP}$: Losses due to surface plasmon polaritons at metal interfaces

In conventional planar OLEDs, a significant portion of generated photons is trapped due to total internal reflection and coupling to SPP modes, limiting external quantum efficiency.

4. Near-Field and Far-Field Calculations

Near-Field Analysis: The spatial distribution of the electromagnetic field inside the device is obtained by solving Maxwell’s equations directly. The power flow is described by the **Poynting vector**:

$$\mathbf{S}=\mathbf{E}\times\mathbf{H} \text{(S3.}\text{7}\text{)}$$

This provides insight into energy confinement, waveguiding, and scattering mechanisms within the OLED stack.

Far-Field Analysis: To evaluate emission characteristics, the near-field data is transformed into the far-field radiation pattern using surface equivalence principles. The far-field electric field is expressed as:

$$\mathbf{E}_{far}\left( \theta,\phi\right)\propto\int_{S} \left[ \left( \hat{n}\times\mathbf{H} \right)-\left( \hat{n}\times\mathbf{E} \right)\times\hat{r} \right]e^{ik\cdot r}dS \text{(S3.}\text{8}\text{)}$$

This transformation enables the calculation of angular emission profiles (polar plots), which represent the distribution of emitted power as a function of angle.

**Momentum Matching and Light Extraction Mechanism**

Efficient light extraction requires converting non-radiative modes into radiative modes. This is achieved by modifying the in-plane wavevector $k_{\parallel}$through structural perturbations.

The momentum matching condition is given by:

$$k_{\parallel}+G=k_{air} \text{(S3.}\text{9}\text{)}$$

where:

- $G = 2\pi/\Lambda is the reciprocal lattice vector$
- $\Lambda is the structural periodicity$

In planar OLEDs, $k_{\parallel}>k_{air}$, preventing light escape. Nanostructures such as IRMLA and GICL can perturb the in-plane wavevector of confined optical components and redistribute optical power toward radiative channels.

The FDTD simulations were used to compare structure-dependent field redistribution, air-side optical power, and far-field angular emission under the defined optical stack and source conditions. These simulations were not used as direct absolute predictions of device EQE or as direct proof of complete local cavity preservation; device-level electrical and optical characteristics are evaluated separately in the main text.
